# Modulation of AMPA receptor surface diffusion restores hippocampal plasticity and memory in Huntington’s disease models

**DOI:** 10.1038/s41467-018-06675-3

**Published:** 2018-10-15

**Authors:** Hongyu Zhang, Chunlei Zhang, Jean Vincent, Diana Zala, Caroline Benstaali, Matthieu Sainlos, Dolors Grillo-Bosch, Sophie Daburon, Françoise Coussen, Yoon Cho, Denis J. David, Frederic Saudou, Yann Humeau, Daniel Choquet

**Affiliations:** 1 Interdisciplinary Institute for Neuroscience, University of Bordeaux, Bordeaux, 33076 France; 2 Interdisciplinary Institute for Neuroscience, Centre National de la Recherche Scientifique (CNRS) UMR 5297, Bordeaux, 33076 France; 3 Department of Biomedicine, KG Jebsen Centre for Research on Neuropsychiatric Disorders, University of Bergen, Jonas Lies vei 91, N-5009 Bergen, Norway; 4 Institut Curie, CNRS, UMR3306, Inserm, U1005, F-91405 Orsay, France; 5 INSERM U894, Center of Psychiatry and Neuroscience, Paris, France, University Paris-Descartes, Paris, 75006 France; 6 Univ. Grenoble Alpes, Grenoble Institut des Neurosciences, GIN, F-38000 Grenoble, France; 7 INSERM, U1216, F-38000 Grenoble, France; 8 Institut de Neurosciences Cognitives et Intégratives d’Aquitaine, University of Bordeaux, Bordeaux, 33000 France; 9 Université Paris-Saclay, Univ. Paris-Sud, Faculté de Pharmacie, CESP, INSERM UMRS1178, Chatenay-Malabry, 92296 France; 10 CHU Grenoble Alpes, F-38000 Grenoble, France; 11 Bordeaux Imaging Center, CNRS UMS 3420, University of Bordeaux, INSERM US04, 33076 Bordeaux, France

## Abstract

Impaired hippocampal synaptic plasticity contributes to cognitive impairment in Huntington’s disease (HD). However, the molecular basis of such synaptic plasticity defects is not fully understood. Combining live-cell nanoparticle tracking and super-resolution imaging, we show that AMPAR surface diffusion, a key player in synaptic plasticity, is disturbed in various rodent models of HD. We demonstrate that defects in the brain-derived neurotrophic factor (BDNF)–tyrosine receptor kinase B (TrkB) signaling pathway contribute to the deregulated AMPAR trafficking by reducing the interaction between transmembrane AMPA receptor regulatory proteins (TARPs) and the PDZ-domain scaffold protein PSD95. The disturbed AMPAR surface diffusion is rescued by the antidepressant drug tianeptine via the BDNF signaling pathway. Tianeptine also restores the impaired LTP and hippocampus-dependent memory in different HD mouse models. These findings unravel a mechanism underlying hippocampal synaptic and memory dysfunction in HD, and highlight AMPAR surface diffusion as a promising therapeutic target.

## Abstract (web summary)

Cognitive decline in Huntington’s disease (HD) may be due to impaired hippocampal synaptic plasticity. In this study the authors show that AMPA receptor surface diffusion, a key player in synaptic plasticity, is deregulated in multiple HD mouse models as a result of impaired BDNF signalling that underlies the memory deficits, and can be pharmacologically rescued.

## Introduction

Cognitive deficits and psychiatric disturbance prior to motor dysfunction have been widely documented in preclinical Huntington’s disease (HD) gene carriers^1,2^. These manifestations have traditionally been attributed to degeneration or death of corticostriatal neurons^3^. However, mounting evidence points to the involvement of deficits in hippocampal synaptic plasticity. This is supported by the findings that hippocampal long-term potentiation (LTP), a major form of synaptic plasticity widely regarded as a molecular basis for learning and memory, is greatly impaired in different categories of HD mouse models at pre- or early-symptomatic stage^4–7^. Moreover, the abnormally increased ability to support long-term depression has also been reported in HD mice^8^. Consistently, behavioral studies reveal deterioration of hippocampal-associated spatial memory in distinct HD murine models, primate model^9^, and patients^10^.

The molecular mechanisms underlying hippocampal synaptic and memory dysfunctions are not well understood but the brain-derived neurotrophic factor (BDNF) signaling pathway seems to play an important role. BDNF is a potent, positive modulator of LTP^11^. The downregulation of its protein production and the imbalance between the expression of its TrkB receptor and the pan-neurotrophin receptor p75 (P75^NTR^) have been implicated in the hippocampal synaptic and memory defects in HD. Indeed, administration of BDNF or P75^NTR^ gene knockdown ameliorates HD-associated synaptic and memory dysfunction^6,12^. However, the signaling mechanisms mediating BDNF modulation of synaptic plasticity and mechanism-based pharmacological treatment strategies remain largely unexplored. This may have significant therapeutic implications as the application of exogenous BDNF is not clinically practical due to its instability in the bloodstream and its inability to cross the blood–brain barrier, and genetic intervention on human subjects may carry ethical issues.

AMPA receptors (AMPARs) are the major excitatory neurotransmitter receptors. The regulated trafficking of AMPARs to and from the synapses is thought to be a key mechanism underlying glutamatergic synaptic plasticity^13–15^. Animal studies reveal that AMPAR trafficking plays a pivotal role in experience-driven synaptic plasticity and modification of behavior^16^. In particular, we recently established that AMPAR surface diffusion is mandatory for expression of LTP^17^. Pathological conditions, such as acute stress or stress hormones, alter AMPAR trafficking and memory encoding processes. Thus monitoring and manipulating synaptic AMPAR trafficking emerges as a useful tool to study cognitive function and dysfunction in animal models. Synaptic delivery of AMPAR involves intracellular trafficking, insertion to the plasma membrane by exocytosis, and lateral diffusion at the neuronal surface^14,17,18^. For many years, endocytosis/exocytosis have been considered to be the main routes for exit and entry of receptors from and to postsynaptic sites, respectively. However, our laboratory and others have established in the past decade that receptor surface diffusion is a key step for modifying receptor numbers at synapses^13,17,19,20^. We have demonstrated that deregulated AMPAR surface diffusion primarily contributes to the impaired LTP in stress/depression models^21^. Most importantly, we found that AMPAR surface diffusion can be pharmacologically modulated by the clinically used antidepressant tianeptine (S 1574, [3-chloro-6-methyl-5, 5-dioxo-6,11-dihydro-(c,f)-dibenzo-(1,2-thiazepine)-11-yl) amino]-7 heptanoic acid), which restores impaired LTP in a stress/depression model^22^. Impaired synaptic plasticity is a common mechanism underlying both cognitive impairment and psychiatric disturbance such as anxiety and depression, the major early-onset symptoms in HD^1,2,23^. Hence, we have examined whether AMPAR surface diffusion is disturbed in HD models, how this is linked with impaired BDNF signaling, and whether pharmacological modulation of AMPAR surface diffusion by tianeptine can serve as a promising therapeutic strategy to improve synaptic and memory dysfunction as well as anxiety/depression-like behavior in HD.

## Results

### AMPAR surface diffusion is deregulated in five HD models

**Fig. 1:**
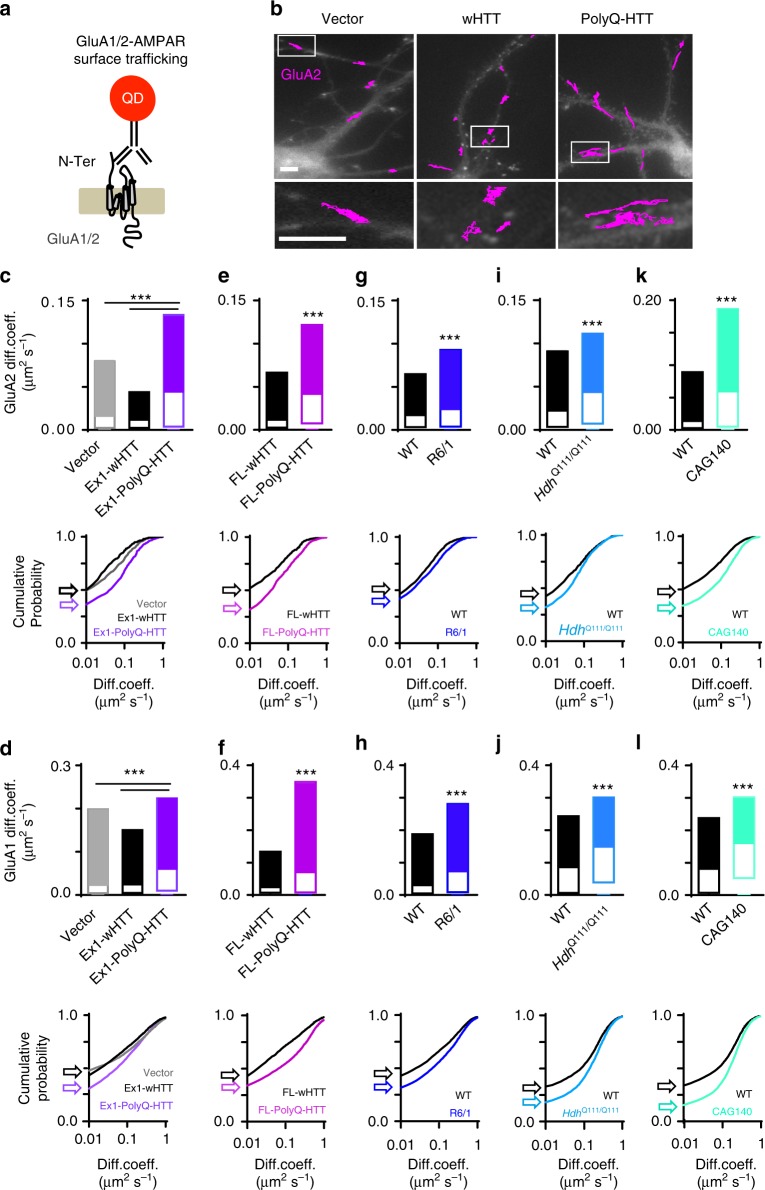
Deregulated GluA2-and GluA1-AMPAR surface diffusion in five complementary HD cellular models. **a** Experimental scheme for endogenous GluA2- and GluA1-AMPAR surface tracking using quantum dot (QD). **b** Typical GluA2-QD trajectories (magenta) in hippocampal neurons expressing vector, exon1-wHTT, and exon1-polyQ-HTT, respectively. Lower panels represent enlarged GluA2-QD trajectories. Scale bars, 10 μm. **c**, **e**, **g, i**, **k** Top panels, GluA2-AMPAR diffusion coefficients (median ± 25–75% interquartile range (IQR)) in rat hippocampal neurons expressing vector, exon1-wHTT, and exon1-polyQ-HTT; *n* = 844, 382, and 695 trajectories, respectively (**c**), in rat hippocampal neurons expressing FL-wHTT or FL-polyQ-HTT; *n* = 603 and 760 trajectories, respectively (**e**), in hippocampal neurons from *R6/1* mice and WT littermate controls; *n* = 1885 and 1994 trajectories, respectively (**g**), in hippocampal neurons from *Hdh*^Q111/Q111^ mice and WT littermate controls; *n* = 1571 and 886 trajectories, respectively (**i**), and in hippocampal neurons from *CAG140* mice and WT littermate controls; *n* = 2939 and 5457 trajectories, respectively (**k**). **d**, **f**, **h**, **j**, **l** Top panels, GluA1-AMPAR diffusion coefficients (median ± 25–75% IQR) in rat hippocampal neurons expressing vector, exon1-wHTT, and exon1-polyQ-HTT; *n* = 9337, 8320, and 10905 trajectories, respectively (**d**), in rat hippocampal neurons expressing FL-wHTT or FL-polyQ-HTT; *n* = 4331 and 6462 trajectories, respectively (**f**), in hippocampal neurons from *R6/1* mice and WT littermate controls; *n* = 18565 and 14195 trajectories, respectively (**h**), in hippocampal neurons from *Hdh*^Q111/Q111^ mice and WT littermate controls; *n* = 5612 and 5844 trajectories, respectively (**j**), and in hippocampal neurons from *CAG140* mice and WT littermate controls; *n* = 8569 and 9170 trajectories, respectively (**l**). Bottom panels, cumulative probability of GluA2 and GluA1 diffusion coefficient of respective top panel. The first point of the probability corresponding to the fraction of immobile receptors with diffusion coefficients ≤0.01 μm^2^ s^−1^ shown by arrows. Note that the cumulative curve shifts toward right indicating an increased GluA2 and GluA1 surface diffusion. Data were from 6 to 12 cells from three separate experiments. Significance was determined by Kruskal–Wallis test followed by Dunn’s multiple comparison test (**c**, **d**) or two-tailed Mann–Whitney test (**e**–**l**); ***P < 0.001

AMPARs are heteromeric proteins composed of different combinations of GluA1, GluA2, GluA3, or GluA4 subunits. GluA1–GluA2 di-heteromers are the most common combination in adult neurons^14^. We thus investigated endogenous GluA2- and GluA1-AMPAR surface diffusion separately using the single nanoparticle tracking approach in which a quantum dot (QD) is coupled to an antibody specific for the extracellular domain of the endogenous GluA2 or GluA1 subunit (Fig. 1a)^22^. We initially used rat primary hippocampal neuronal cultures co-transfected with homer1c-GFP and exon1 mutant huntingtin, which contains 69 polyglutamine expansion (exon1-polyQ-HTT), with exon1 wild-type (WT) huntingtin with 17 polyglutamine (exon1-wHTT) and empty vector as controls^24^. Compared to empty vector and exon1-wHTT, expression of exon1-polyQ-HTT significantly increased the surface diffusion of GluA2- and GluA1-AMPAR (Fig. 1c, d, top panel). We confirmed the co-transfection efficiency of green fluorescent protein (GFP) and HTT fused with hemagglutinin (HA) tag (ratio 1:4). All GFP-expressing cells were successfully transfected with HTT as revealed by HA staining (Supplementary Fig. 1a, 1b). We next used rat primary hippocampal neuronal cultures co-transfected with GFP and full-length mutant huntingtin, which contains 75 polyglutamine expansion (FL-polyQ-HTT), with FL WT huntingtin with 17 polyglutamine (FL-wHTT) as controls. Compared to FL-wHTT-expressing cells, GluA2- and GluA1-AMPAR surface mobility was significantly increased in FL-polyQ-HTT-expressing cells (Fig. 1e, f, top panel). To avoid possible transfection artifacts, we next used primary hippocampal neurons from *R6/1* heterozygous transgenic mice, which overexpress the first exon of human HTT with 115 polyQ and represent a fast model of HD. Similarly, an increase in GluA2- and GluA1-AMPAR surface diffusion was observed in neurons from *R6/1* mice compared to WT littermate controls (Fig. 1g, h, top panel). Furthermore, to circumvent overexpression artifacts and to better mimic the genetic situation in patients, we used neurons from homozygous *Hdh*^Q111/Q111^ knock-in (KI) mouse, in which polyQ repeats are directly engineered into the mouse HTT genomic locus and wHTT/polyQ-HTT is expressed at endogenous levels. Consistently, neurons from *Hdh*^Q111/Q111^ KI mouse also displayed marked increase in GluA2- and GluA1-AMPAR surface diffusion compared to WT littermates (Fig. 1i, j, top panel). Finally, we used *CAG140* heterozygous KI mice. Heterozygous mice are highly relevant to the disease, as the majority of HD patients are heterozygous^3^. In line with the above-mentioned findings, GluA2- and GluA1-AMPAR surface diffusion was significantly increased in *CAG140* mice compared to WT littermates (Fig. 1k, l, top panel). These changes were partially due to a decreased fraction of immobile GluA2- and GluA1-AMPAR (surface diffusion ≤0.01 µm^2^ s^−1^) (Fig. 1c–l, bottom panels). Cumulative distributions of diffusion coefficients shift toward the right in all HD models, indicating an increased GluA2- and GluA1 AMPAR surface diffusion.

### AMPAR level and synaptogenesis are unaltered in HD models

We next evaluated the surface and total GluA2 expression in different HD models. No significant difference was found in surface and total GluA2 expression in FL-wHTT-/polyQ-HTT-expressing neurons (Supplementary Fig. 1c–f) neither in *R6/1* mouse models (Supplementary Fig. 1g–j). We further assessed the synapse numbers and did not find significant difference between FL-wHTT and polyQ-HTT-expressing neurons (Supplementary Fig. 1k, 1l). Altogether, these experiments indicate that disturbance of AMPAR surface diffusion occurs independently of AMPAR protein expression levels and synaptogenesis.

### BDNF synthesis and transport are decreased in HD models

**Fig. 2:**
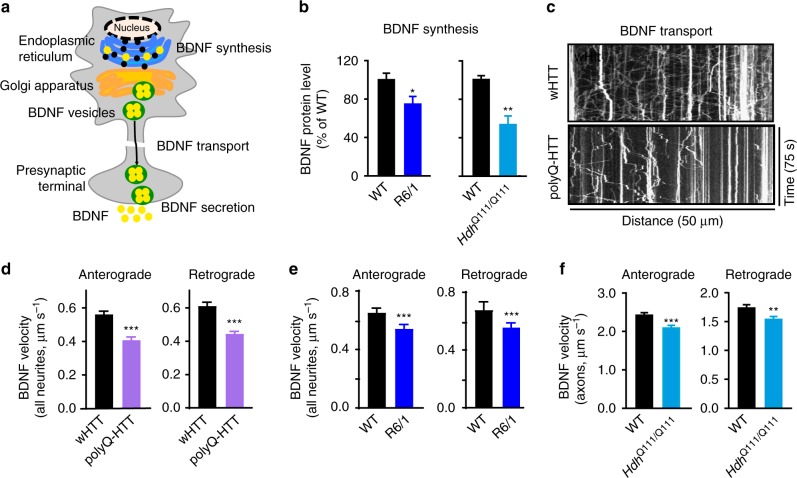
BDNF synthesis and intracellular transport were impaired in various HD models. **a** Schematic diagram showing that BDNF is modulated at synthesis, transport, and secretion levels. **b** Hippocampal BDNF protein level determined by ELISA in *R6/1* and *Hdh*^Q111/Q111^ mice; values are mean ± s.e.m (% of WT); *n* = 21 and 14 mice for WT and *R6/1*; *n* = 6 and 9 mice for WT and *Hdh*^Q111/Q111^, respectively. **c** Representative kymographs of intracellular transport of BDNF-containing vesicles (white trajectories) in a neurite (50 μm from soma) over 75 s in wHTT- and polyQ-HTT-expressing rat hippocampal neurons. The velocity of BDNF transport was reflected by the slope of trajectories (moving distance against time). **d**–**f** Anterograde and retrograde BDNF transport velocity in all neurites of wHTT- and polyQ-HTT-expressing rat hippocampal neurons (**d**) and hippocampal neurons from *R6/1* mouse line (**e**) and in the axon of hippocampal neurons from *Hdh*^Q111/Q111^ mouse line (**f**); values are median ± 95% c.i. (**d**, **e**) or mean ± s.e.m (**f**); *n* = 5569, 5656, 5227, and 5706 trajectories for anterograde and retrograde wHTT and polyQ-HTT, respectively; *n* = 1424, 1710, 1376, and 1487 trajectories for anterograde and retrograde WT and *R6/1*, respectively; *n* = 236, 261, 194, and 256 trajectories for anterograde and retrograde WT and *Hdh*^Q111/Q111^, respectively. Significance was determined by unpaired two-tailed Student’s *t* test (**b**, **f**) or two-tailed Mann–Whitney test (**d**, **e**); *P < 0.05, **P < 0.01, ***P < 0.001

BDNF is a prominent positive modulator of LTP^11^, which has been proposed to induce the delivery of AMPARs to the synapse under basal conditions^25^. However, it is not known whether and how AMPAR surface diffusion is modulated by BDNF signaling and whether it plays a role in HD pathogenesis. We thus asked whether deficient BDNF signaling could account for the aberrant AMPAR surface diffusion in HD mouse models. We first characterized changes in the protein level of BDNF in HD mice. Consistent with a previous report^6^, using enzyme-linked immunosorbent assay (ELISA), we observed a significant decrease in the protein level of BDNF in the hippocampus of 10-week-old male *R6/1* and *Hdh*^Q111/Q111^ mice compared to respective littermate controls (Fig. 2b). We next studied BDNF intracellular transport in 3 complementary HD cellular models, as data on the BDNF intracellular transport in the hippocampus of HD mice are still lacking. Neurons expressing polyQ-HTT exhibited slower anterograde and retrograde BDNF intracellular transport relative to wHTT-expressing neurons (Fig. 2c, d). Similarly, *R6/1* and *Hdh*^Q111/Q111^ mouse hippocampal neurons exhibited slower transport compared to respective WT littermate controls (Fig. 2e, f, respectively). Note that we observed a slower BDNF velocity in neurites (Fig. 2d, e) as compared to axons in hippocampal neurons (Fig. 2f), which is consistent with previous studies in cortical neurons^26,27^. Altogether, these data suggested that reduced BDNF protein production and impaired intracellular transport are common features of different categories of HD models.

### Impaired BDNF-TrkB signaling impacts AMPAR surface diffusion

Next, we dissected the potential signaling mechanism by which BDNF modulates AMPAR surface diffusion in HD models. BDNF is known to bind to TrkB receptors, leading to the activation of CaMKII^11^, which is known to regulate AMPAR surface diffusion via impacting the interaction between TARP-2 (stargazin) and PSD95 during plasticity^28^. Active CaMKII phosphorylated at threonine 286 (T286) is reported to be reduced in the hippocampus of *Hdh*^Q111/Q111^ mouse models^12^. We also detected the decrease in CaMKII activity in a HD cellular model by co-transfecting rat hippocampal neurons with FL-wHTT/FL-polyQHTT and a fluorescence resonance energy transfer (FRET)-based CaMKIIα, named REACH-CaMKII. The amino and carboxy termini of REACH-CaMKII are labeled with the FRET pair of monomeric enhanced GFP (mEGFP) and resonance energy-accepting chromoprotein (REACh), a non-radiative yellow fluorescent protein variant^29^. The activation of REACh-CaMKII associated with T286 phosphorylation changes the conformation of CaMKIIα to the open state in which its kinase domain is exposed, thereby decreasing FRET and increasing the fluorescence lifetime of mEGFP (Supplementary Fig. 2a). Rat hippocampal neurons transfected with GFP-PSD95 alone were used as negative control. GFP-PSD95-expressing cells displayed long lifetime (≥2.4 ns) in both dendritic puncta and shaft indicating no FRET. FL-wHTT- and FL-polyQ-HTT-expressing cells both exhibited FRET revealed by shorter lifetime than GFP-PSD95-expressing cells, indicating activated CaMKIIα. However, the REACh-CaMKIIα lifetime in dendritic puncta (Supplementary Fig. 2c) and shaft (Supplementary Fig. 2d) in FL-polyQ-HTT-expressing cells are significantly lower than in FL-wHTT-expressing cells, indicating stronger FRET and thus weaker CaMKIIα activity.

**Fig. 3:**
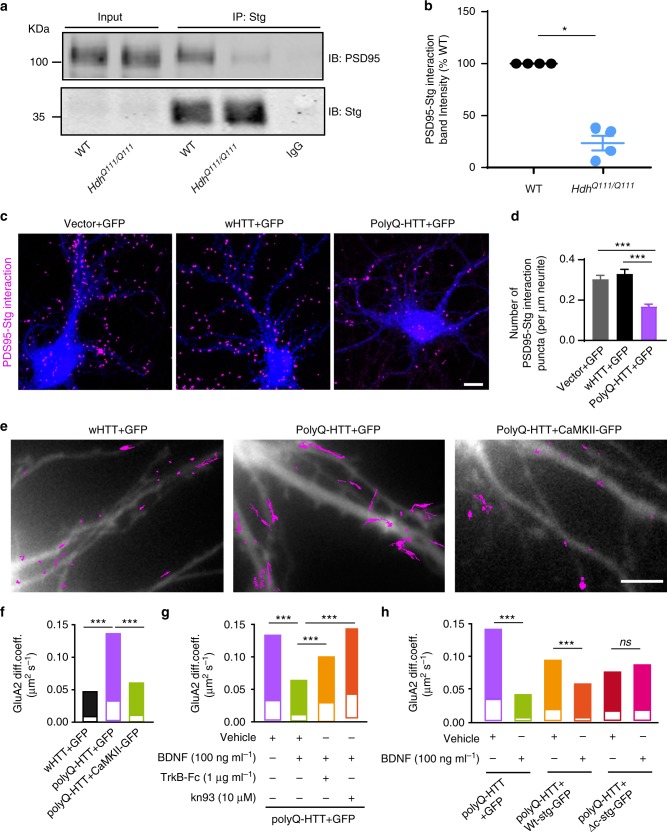
Impaired BDNF-TrkB signaling contributes to the deregulated AMPAR surface diffusion by disrupting stargazin–PSD95 interaction in HD models. **a** Representative blots of co-immunoprecipitation (Co-IP) of PSD95 and stargazin complexes. **b** Quantified densitometry of PSD95–Stg interaction band, which is expressed as percentage relative to WT; *n* = 4 for WT and *Hdh*^Q111/Q111^ mice. **c** Representative images of PSD95–Stg interaction determined by DUOLINK in situ Proximity Ligation Assay, the number of the magenta puncta indicates the strength of the interaction. Scale bar, 10 μm. **d** Quantification of PSD95–Stg interaction strength in different groups for PLA, *n* = 35, 33, and 28 cells for Vector, wHTT, and polyQ-HTT groups, respectively. **e** Typical GluA2-QD trajectories (magenta) in hippocampal neurons co-expressing FL-wHTT/polyQ-HTT and GFP or FL-polyQ-HTT and CamKII-GFP, respectively. Scale bar, 10 μm. **f**–**h** GluA2-AMPAR diffusion coefficients in rat hippocampal neurons co-expressing FL-wHTT/polyQ-HTT and GFP or FL-polyQ-HTT and CamKII-GFP; *n* = 656, 685, and 349 trajectories, respectively (**f**), in neurons co-expressing FL-polyQ-HTT and GFP and treated with Vehicle, BDNF, TrkB-Fc plus BDNF, or kn93 plus BDNF; *n* = 1649, 1742, 480, and 1380 trajectories, respectively (**g**), and in vehicle- or BDNF-treated neurons co-expressing FL-polyQ-HTT and GFP or GFP-fused wild-type stargazin (Wt-stg-GFP) or ΔC stg, in which the binding domain to PDZ-domain scaffold protein was deleted; *n* = 495, 568, 376, 300, 573, and 498 trajectories, respectively (**h**). Diffusion coefficients were shown as median ± 25–75% IQR; Values are mean ± s.e.m (**b**, **d**); data are representative of a minimum of three independent experiments. Significance was determined by one-sample *t* test (**b**), one-way ANOVA followed by Tukey’s multiple comparison test (**d**), or Kruskal–Wallis test followed by Dunn’s multiple comparison test (**f**–**h**); *P < 0.05, ***P < 0.001, ns no significance

The CaMKII-induced AMPAR immobilization requires the interaction between stargazin and PSD95^22,28^. We thus examined whether stargazin–PSD95 interaction was weakened in different HD models. We first used co-immunoprecipitation (Co-IP) and demonstrated that PSD95–stargazin interaction was impaired in the brain tissue from *Hdh*^Q111/Q111^ mice relative to WT littermates (Fig. 3a, b, Supplementary Fig. 6a). We further performed DUOLINK in situ proximity ligation assay (PLA), which allows for endogenous detection of protein interactions at the single molecule level, in hippocampal neurons co-transfected with GFP and vector, wHTT, or polyQ-HTT. Consistently, we found that the PSD95–stargazin interaction was also markedly decreased in polyQ-HTT-expressing neurons compared with vector and wHTT controls (Fig. 3c, d).

We reasoned that, if reduced CaMKII activity is responsible for aberrant AMPAR surface trafficking, then overexpression of constitutively active CaMKII should be able to rescue the FL-polyQ-HTT-induced increase in AMPAR surface diffusion. This is indeed what we observed (Fig. 3e, f). We assumed that, if reduced CaMKII activity results from impaired BDNF-TrkB signaling pathway, then the application of exogenous BDNF should have similar effect. As expected, the application of exogenous BDNF similarly restored a lower GluA2-AMPAR surface diffusion (Fig. 3g, green bar). Moreover, BDNF application could also decrease the AMPAR surface mobility in WT neurons (Supplementary Fig. 3). This rescue effect of BDNF requires the activation of TrkB and CaMKII as this effect was completely blocked by the addition of the BDNF scavenger TrkB-Fc or CaMKII inhibitor kn93 (Fig. 3g, orange and red bars, respectively). This indicates that BDNF-TrkB-CaMKII signaling pathway plays a key role in stabilizing surface AMPARs. We next examined the role of the interaction between stargazin and PSD95 in mediating BDNF’s effects on AMPAR surface diffusion by expressing ΔC stargazin (ΔC Stg), in which the interaction domain with PDZ-domain scaffold proteins was deleted. In ΔC-Stg but not WT stargazin-expressing neurons, administration of BDNF failed to reduce GluA2-AMPAR surface diffusion (Fig. 3h). These data suggest that impaired BDNF-TrkB-CaMKII signaling contributes to the disturbance of AMPAR surface diffusion, possibly by reducing interaction between TARP family protein and PDZ-domain scaffold proteins in the hippocampus of HD models.

### Tianeptine improves BDNF level and transport in HD models

**Fig. 4:**
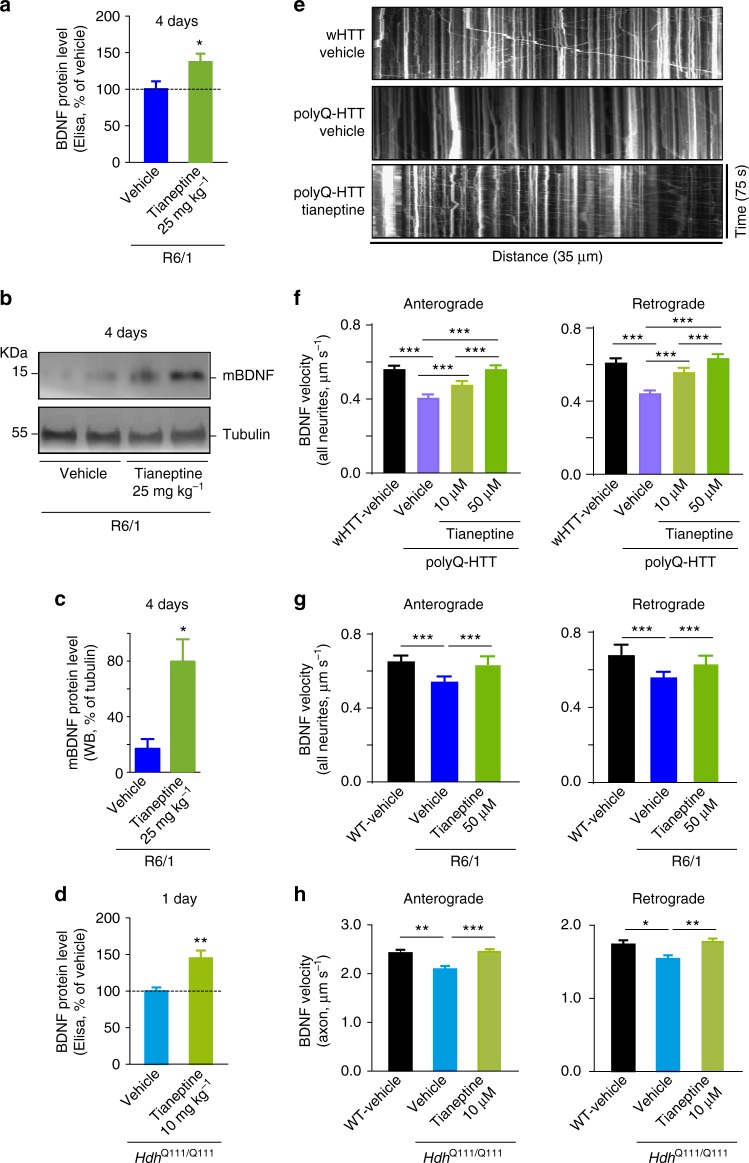
Tianeptine rescues BDNF protein level and transport in complementary HD models. **a**–**c** *R6/1* mice were treated with vehicle (saline) or tianeptine (25 mg kg^−1^, i.p. daily) for 4 days. Hippocampal BDNF protein level was assessed using ELISA (**a**); *n* = 14 and 13 mice for vehicle- and tianeptine-treated *R6/1* group, respectively. Mature BDNF (mBDNF) and tubulin were analyzed by immunoblot (**b**); quantified densitometry of mBDNF was expressed as percentage relative to tubulin (**c**); *n* = 9 and 7 mice for vehicle- and tianeptine-treated *R6/1* group, respectively. **d** *Hdh*^Q111/Q111^ mice received one injection of saline or tianeptine (10 mg kg^−^^1^, i.p.). Hippocampal BDNF protein level was evaluated using ELISA; *n* = 9 and 8 mice for vehicle- and tianeptine-treated *Hdh*^Q111/Q111^ group, respectively. **e** Representative kymographs of intracellular transport of BDNF-containing vesicles (white trajectories) in a neurite (35 μm from soma) over 75 s in vehicle- or tianeptine-treated rat hippocampal neurons expressing wHTT or polyQ-HTT. **f**–**h** Anterograde and retrograde BDNF transport velocity in all neurites of vehicle- or tianeptine-treated wHTT- and polyQ-HTT-expressing rat hippocampal neurons (**f**), of hippocampal neurons from vehicle- or tianeptine-treated *R6/1* mice and WT littermates (**g**), and in the axons of hippocampal neurons from vehicle- or tianeptine-treated *Hdh*^Q111/Q111^ and WT mice (**h**); *n* = 5569, 5656, 3339, 5737, 5227, 5706, 3190, and 5663 trajectories for anterograde and retrograde BDNF velocity in wHTT-vehicle, polyQ-HTT-vehicle, and polyQ-HTT-tianeptine (10 and 50 μM) neurons, respectively; *n* = 1424, 1710, 1512, 1376, 1487, and 1238 trajectories for anterograde and retrograde velocity in WT-vehicle, *R6/1*-vehicle, and *R6/1*-tianeptine (50 μM) neurons, respectively; *n* = 236, 261, 432, 194, 256, and 357 trajectories for anterograde and retrograde velocity in WT-vehicle, *Hdh*^Q111/Q111^ -vehicle, and *Hdh*^Q111/Q111^ -tianeptine (10 μM) neurons, respectively. Values are mean ± s.e.m (**a**, **c**, **d**, **h**) or median ± 95% c.i. (**f**, **g**). Data are from three independent experiments. Significance was determined by unpaired two-tailed Student’s *t* test (**a**, **c**, **d**), Kruskal–Wallis test followed by Dunn’s multiple comparison test (**f**, **g**), or one-way ANOVA followed by Tukey multiple comparison test (**h**); *P < 0.05, **P < 0.01, ***P *<* 0.001

BDNF is not a good candidate for HD treatment due to its instability and difficulties to cross the blood–brain barrier^30–32^. An alternative approach, therefore, is to elevate endogenous BDNF protein or trafficking using other exogenous agents. Our previous work showed that the antidepressant tianeptine modulates AMPAR surface diffusion and improved LTP in stress/depression models^22^. It has also been reported that chronic tianeptine treatment increased BDNF protein level in various rodent brain structures^33,34^. However, the effect of tianeptine on BDNF intracellular trafficking is not known and it is unclear whether tianeptine modulates BDNF signaling in HD models. We thus examined the effect of tianeptine on BDNF protein production as well as intracellular trafficking in different HD models. Hippocampal BDNF protein production was evaluated using ELISA and western blot methods in *R6/1* and *Hdh*^Q111/Q111^ mice at 10–12 weeks of age. Because at this age, *R6/1* and *Hdh*^Q111/Q111^ mice were reported to show LTP defects and *R6/1* mice gradually develop cognitive deficits^4,6^. We found that the reduced hippocampal BDNF protein levels in *R6/1* (Fig. 4a–c, Supplementary Fig. 6b) and in *Hdh*^Q111/Q111^ mice (Fig. 4d) were both significantly improved by tianeptine administration (25 mg kg^−1^, intraperitoneally (i.p.) daily for 4 days for *R6/1* mice; and 10 mg kg^−^^1^, once for *Hdh*^Q111/Q111^ mice). Note that a single tianeptine injection at 10 mg kg^−1^ was inefficient for *R6/1* mice (data not shown), which may be due to the more severe phenotypes in this mouse model^35^. We next examined tianeptine effect on BDNF intracellular trafficking in three different HD cellular models. The application of tianeptine fully rescued the velocity of BDNF anterograde and retrograde transport in polyQ-HTT-expressing neurons (Fig. 4e, f) as well as in hippocampal neurons from *R6/1* (Fig. 4g) and *Hdh*^Q111/Q111^ mice (Fig. 4h). In addition, tianeptine also augmented BDNF intracellular trafficking in wHTT-expressing neurons and in neurons from WT control for *Hdh*^Q111/Q111^ mice (Supplementary Fig. 4). These data suggest that tianeptine regulates hippocampal BDNF signaling at least at two levels, namely, BDNF protein production and intracellular transport.

### Tianeptine rescues BDNF and AMPAR trafficking deficits via BDNF-TrkB pathway

**Fig. 5:**
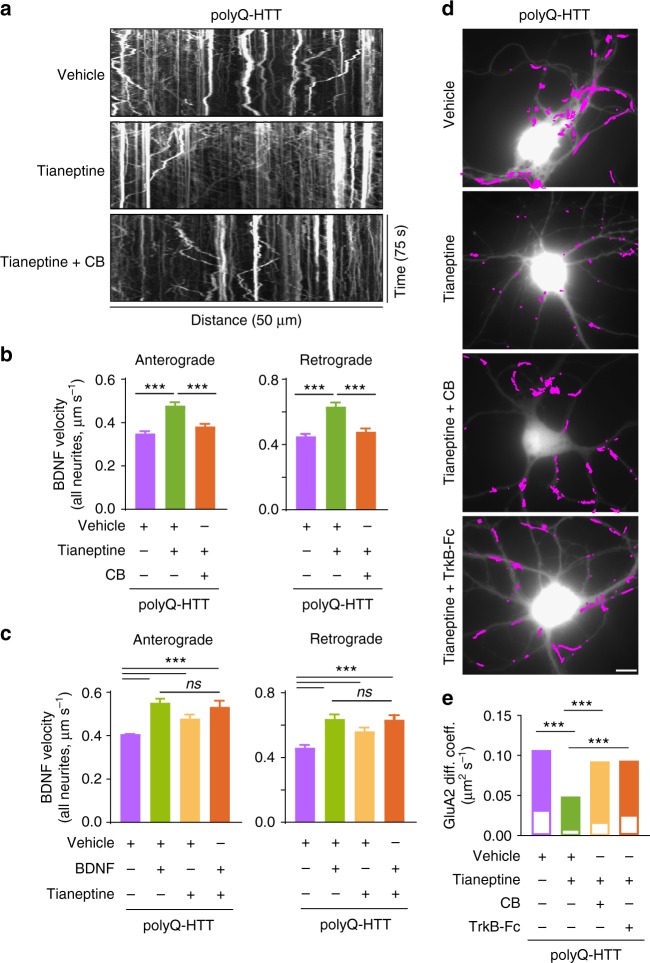
Tianeptine’s effect on BDNF intracellular transport and AMPAR surface diffusion is mediated by BDNF-TrkB signaling pathway. **a** Representative kymographs of intracellular transport of BDNF-containing vesicles (white trajectories) in a neurite (50 μm from soma) over 75 s in polyQ-HTT-expressing rat hippocampal neurons treated with vehicle, tianeptine (50 μM), or Cyclotraxin-B (CB) (1 μM) plus tianeptine (50 μM). **b**, **c** Anterograde and retrograde BDNF transport velocity in all neurites of polyQ-HTT-expressing rat hippocampal neurons treated with vehicle, tianeptine (50 μM), or CB (1 μM) plus tianeptine (50 μM) (**b**) or treated with vehicle, BDNF (100 ng ml^−^^1^), tianeptine (10 μM), or BDNF (100 ng ml^−1^) plus tianeptine (10 μM) (**c**); values are median ± 95% c.i. (**b**, **c**); *n* = 4322, 4017, 4199, 4354, 3887, and 3954 trajectories for anterograde and retrograde velocity in polyQ-HTT-expressing neurons treated with vehicle, tianeptine, and CB plus tianeptine, respectively (**b**); *n* = 3505, 3382, 3339, 2099, 3346, 3174, 3190, and 2022 trajectories for anterograde and retrograde velocity in polyQ-HTT-expressing neurons treated with vehicle, BDNF, tianeptine, and BDNF plus tianeptine, respectively (**c**). **d** Typical GluA2-QD trajectories (magenta) in polyQ-HTT-expressing rat hippocampal neurons, treated with vehicle, tianeptine (50 μM), CB (1 μM) plus tianeptine (50 μM), or TrkB-Fc (1 μg ml^−1^) plus tianeptine (50 μM). Scale bar, 10 μm. **e** GluA2-AMPAR diffusion coefficients in FL-polyQ-HTT-expressing rat hippocampal neurons treated with vehicle, tianeptine (50 μM), CB (1 μM), plus tianeptine (50 μM), or TrkB-Fc (1 μg ml^−1^) plus tianeptine (50 μM); data are shown as median ± 25–75% IQR; *n* = 601, 535, 708, and 556 trajectories for 4 groups, respectively. Data are representative of a minimum of three independent experiments. Significance was assessed by Kruskal–Wallis test followed by Dunn’s multiple comparison test (**b**, **c**, **e**); ***P *<* 0.001; ns not significant

To further clarify the functional mechanism of tianeptine, we examined whether the tianeptine-induced increase in BDNF intracellular trafficking could be prevented by a selective TrkB receptor inhibitor, Cyclotraxin-B (CB), which is a small inhibitor peptide mimicking the reverse turn structure of the variable region III that protrudes from the core of BDNF^36^. Indeed, tianeptine (50 μM) induced improvement of anterograde and retrograde BDNF intracellular transport was fully blocked by pre-incubation with CB (1 μM) (Fig. 5a, b). This suggested that tianeptine’s effect on BDNF intracellular trafficking is likely mediated through TrkB receptor. Since BDNF is not the sole ligand for TrkB receptor, we then postulated that, if tianeptine influences BDNF intracellular trafficking through BDNF signaling rather than working in parallel, then addition of exogenous BDNF should be able to occlude tianeptine’s effect. Indeed, the administration of BDNF (100 ng ml^−1^) similarly rescued the decreased BDNF intracellular trafficking induced by polyQ-HTT and the combination of BDNF and tianeptine did not exhibit additive effect (Fig. 5c). These data indicate that tianeptine affects BDNF intracellular trafficking possibly through BDNF-TrkB signaling pathway. We next asked whether tianeptine is also able to restore AMPAR surface traffic and whether this effect is mediated by TrkB receptors. The application of tianeptine significantly slowed down AMPAR surface diffusion in polyQ-HTT-expressing neurons, an effect fully blocked by the TrkB receptor inhibitor CB and TrkB-Fc (Fig. 5d, e). Collectively, these data suggest that the tianeptine effect on BDNF intracellular trafficking and AMPAR surface diffusion is mediated by BDNF-TrkB signaling pathway.

### Tianeptine restores AMPAR dynamics after LTP induction

**Fig. 6:**
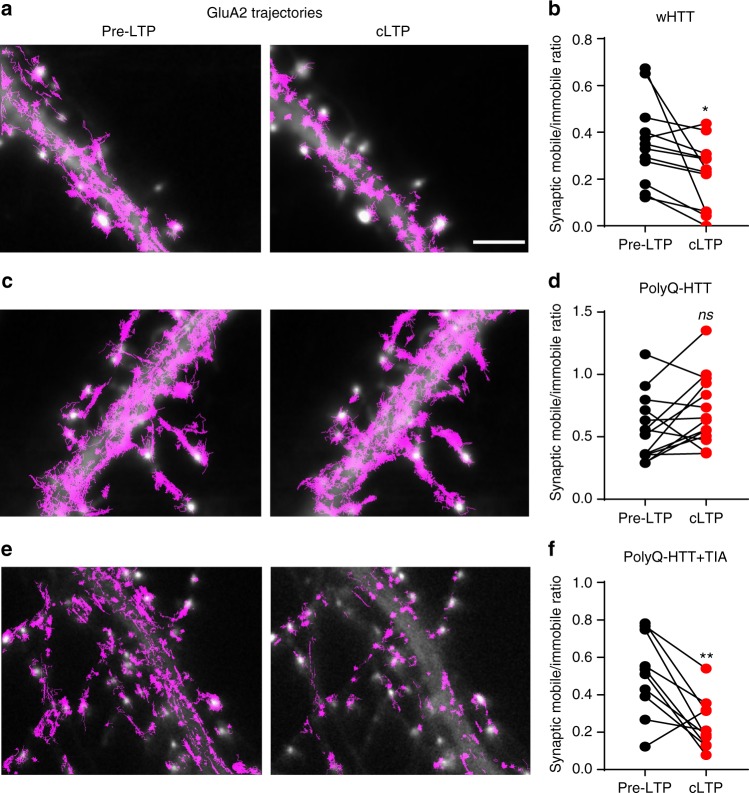
AMPAR fails to stabilize on the neuronal surface after LTP stimulation, an effect rescued by tianeptine (TIA) treatment in an HD cellular model. **a**, **c**, **e** Typical GluA2 trajectories (magenta) before and after cLTP induction in FL-wHTT-expressing neurons without TIA treatment (**a**), FL-polyQ-HTT-expressing neurons without TIA treatment (**c**), and FL-polyQ-HTT-expressing neurons with TIA treatment (10 μM) (**e**). Scale bar, 5 μm. **b**, **d**, **f** The ratio of mobile to immobile fraction of the diffusion coefficient (*D*) at synapses before and after cLTP induction in FL-wHTT-expressing neurons without TIA treatment (**b**), FL-polyQ-HTT-expressing neurons without TIA treatment (**d**), and FL-polyQ-HTT-expressing neurons with TIA treatment (10 μM) (**f**). Data were from 10 to 14 neurons from three separate experiments. Immobile fraction was identified as the proportion of receptors with *D* ≤ 0.01 μm^2^ s^−1^ while mobile fraction with *D* > 0.01 μm^2^ s^−1^. Paired *t* test was used; *P < 0.05; **P < 0.0;1; ns not significant

The increased AMPAR surface diffusion in basal conditions in HD models prompted us to ask whether this could potentially lead to abnormal AMPAR surface stabilization during activity-dependent synaptic plasticity, such as LTP. Indeed, on the one hand, it has been shown that activity-dependent synaptic potentiation is associated with immobilization and subsequent accumulation of AMPARs at synapses and that AMPAR surface diffusion is mandatory for LTP^17^. On the other hand, polyQ expansion of HTT is associated with impaired LTP^4–7^. We thus examined AMPAR surface diffusion before and after 3-min chemical LTP (cLTP) stimuli^37^ (300 μM Glycine, 1 μM Picrotoxin, without Mg^2+^) in rat hippocampal neurons overexpressing FL-wHTT or FL-polyQ-HTT using a super-resolution imaging method, universal point accumulation for imaging in nanoscale topography (uPAINT)^38^. uPAINT is not only able to generate super-resolved images but also provides dynamic information with large statistics revealing localization-specific diffusion properties of membrane biomolecules. Endogenous GluA2-AMPARs were tracked with ATTO 647 labeled anti-extracellular GluA2 antibody and sorted into two groups according to their diffusion coefficient (immobile, Log (*D*) = < −2; mobile, Log (*D*) > −2). In FL-wHTT-expressing neurons, we observed a decrease in the ratio of mobile to immobile AMPAR after cLTP stimuli relative to basal condition (Pre-LTP), reflecting an immobilization of surface AMPARs (Fig. 6a, b). In contrast, the ratio of mobile to immobile AMPAR in FL-polyQ-HTT-expressing neurons was not significantly different before and after LTP stimuli (Fig. 6c, d). The effect was fully rescued by the application of tianeptine (10 µM) (Fig.6e, f). These data suggest that AMPARs fail to stabilize at the neuronal surface after LTP stimuli in HD models, which could explain, at least in part, the defects in the potentiation of AMPAR-mediated synaptic transmission in HD. The rescue effect of tianeptine prompted us to further evaluate its beneficial effects on LTP deficits in ex vivo conditions.

**Fig. 7:**
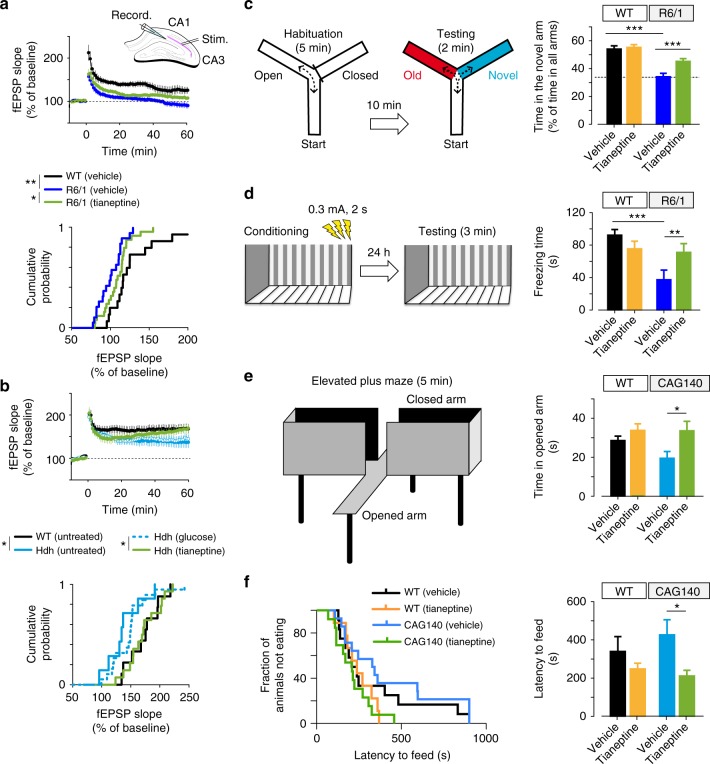
Tianeptine restores hippocampal LTP and memory and ameliorates anxiety/depression like behavior in complementary HD mouse models. **a**, **b** Field EPSPs (fEPSPs) were recorded in CA1 region containing acute slices of differently treated *R6/1* (**a**) and *Hdh*^Q111/Q111^ mice (**b**) following theta-burst stimulation of the Schaffer collaterals. The percentages of potentiation during last 10 min of each recordings were plotted in cumulative probability curves (bottom panels); *n* = 16, 22, and 26 slices for vehicle-treated WT and *R6/1* mice and tianeptine-treated *R6/1* mice; *n* = 10, 8, 20, 15 slices for untreated WT, *Hdh*^Q111/Q111^ mice, and glucose- and tianeptine-treated *Hdh*^Q111/Q111^ mice. **c**, **d** Hippocampus-dependent memory was examined using Y-maze (**c**) and contextual fear conditioning paradigm (**d**) in vehicle- or tianeptine-treated *R6/1* and WT littermate mice. **c** Left, schematic diagram for Y-maze; right, percentage of time spent by mice in novel arms to that in total arms during 2-min testing time. **d** Left, schematic diagram for contextual fear conditioning; right, freezing time during 3-min testing time; *n* = 25, 28, 33, and 32 mice (**c**) and *n* = 10, 10, 10, and 12 mice (**d**) for vehicle- and tianeptine-treated WT and *R6/1* mice. **e**, **f** Anxiety/depression-like behaviors were evaluated with elevated plus maze (EPM) (**e**) and novelty-suppressed feeding (NSF) paradigm (**f**) in HD *CAG140* knock-in mice and WT littermates. **e** Left, schematic diagram for EPM; right, time spent in open arms in EPM, which is an anxiety index. **f** Values plotted were cumulative survival of animals that did not eat over 15 min (left) or mean of latency to feed in seconds (right). The latency to begin eating is an index of anxiety/depression-like behavior; *n* = 12, 9, 14, and 13 mice for vehicle- and tianeptine-treated WT and *CAG140* mice (**e**, **f**). Data values are mean ± s.e.m (**c**–**f**). Significance was assessed by Mann–Whitney/Wilcoxon’s test (**a**, **b**), and two-way ANOVA followed by Tukey multiple comparison test with genotype and treatment as the between-subjects factors (**c**–**f**); *P < 0.05, **P < 0.01, ***P *<* 0.001

### Tianeptine restores hippocampal LTP and memory in HD models

BDNF-TrkB signaling and AMPAR surface diffusion are critically involved in hippocampal plasticity and learning and memory^11,14,17^. We thus asked whether tianeptine could rescue the impaired hippocampal LTP and hippocampal-dependent memory in three different mouse models of HD. Besides male heterozygous *R6/1* transgenic mice and homozygous *Hdh*^Q111/Q111^ KI mice, we employed a third mouse model, male *CAG140* heterozygous KI mice, for behavior test. *CAG140* KI mice carry 140 polyQ and thus have earlier onset of symptoms than *Hdh*^Q111/Q111^ KI mice. Only male HD mice were used throughout the paper in order to eliminate the possible influence of gender differences. The field excitatory postsynaptic potentials (fEPSPs) were recorded from mouse hippocampal CA1 region following Schaffer collateral stimulation (Fig. 7a, b). *R6/1* transgenic mice and *Hdh*^Q111/Q111^ KI mice were used at the age of 10–12 weeks. *R6/1* mice showed a decrease in LTP of fEPSP slope compared to WT littermate controls, which was partially rescued by chronic treatment with tianeptine at 25 mg kg^−1^ (i.p. daily for 8 weeks) (Fig. 7a) but not tianeptine at 10 mg kg^−1^ (i.p. daily for 8 weeks) (data not shown). This suggested a dose-dependent effect of tianeptine. Very similar results were obtained in *Hdh*^Q111/Q111^ mice, in which LTP defects were normalized by a single injection of tianeptine (10 mg kg^−1^) (Fig. 7b).

The restorative effect of tianeptine on LTP in HD mice raises the question of whether it can also rescue HD-related hippocampus-dependent cognitive impairments. In order to test early therapeutic intervention, we started to administer saline/tianeptine (10 mg kg^−1^, i.p. daily) to *R6/1* and WT littermate mice from 4 weeks of age, when the mice do not typically present cognitive deficits^4^. At 12 weeks of age, the mice were subjected to open field test, Y-maze, and contextual fear conditioning. The latter two tasks are hippocampal-dependent memory tasks, respectively, based on novelty attractiveness and associated threat^39^. Vehicle-treated *R6/1* mice spent much less time in the novel arm than vehicle-treated WT mice, suggesting that *R6/1* mice have impaired spatial working memory (Fig. 7c). Interestingly, tianeptine administration improved Y-maze performance of *R6/1* mice but not that of WT mice. This improvement is not due to a change in moving velocity, as vehicle- and tianeptine-treated *R6/1* mice had similar moving velocity in open field (Supplementary Fig. 5a). Contextual fear conditioning, assessed by measuring the freezing behavior a mouse typically exhibits when re-exposed to a context in which a mild foot shock was beforehand delivered, reflects hippocampal-dependent memory^40^. Vehicle-treated *R6/1* mice exhibited less freezing in the contextual fear test compared to vehicle-treated WT littermates, indicating a worse memory, which was rescued by tianeptine treatment (Fig. 7d). Similar to the spatial working memory tested in Y-maze, no beneficial effect was observed in tianeptine-treated WT mice. We thus propose that tianeptine specifically rescued the hippocampal-dependent memory of *R6/1* mice.

As HD mice also typically present an anxiety/depression-like phenotype, we asked whether chronic tianeptine treatment would correct the anxiety/depression-like phenotype in *CAG140* heterozygous KI mice. The anxiety/depression-like behavior of *CAG140* mice was assessed using the elevated plus maze (EPM) paradigm and novelty suppressed feeding (NSF) paradigm. Anxiety-like phenotypes are characterized by decreased time spent in open arms in EPM or an increase in latency to feed in NSF, which we observed in 6-month-old *CAG140* mice (data not shown). Here we specifically investigated the early intervention therapy in HD and treated *CAG140* mice starting from 3 months of age, when the anxiety/depression-like phenotype is not fully established (compared to 6-month-old mice, data not shown) and tested at 4 months of age. We found that, in comparison to vehicle-treated *CAG140* mice, chronically tianeptine-treated *CAG140* mice spent significantly more time in open arms in EPM (Fig. 7e), while their locomotor activity revealed by ambulatory distance was not significantly affected (Supplementary Fig. 5b). The treatment also markedly decreased the latency to feed in NSF (Fig. 7f) without altering the home food consumption (Supplementary Fig. 5c). In contrast, tianeptine did not significantly alter the behavioral phenotype of WT mice, suggesting that chronic tianeptine treatment also specifically improves the anxiety/depression-like behavior in HD mice.

## Discussion

AMPAR surface diffusion plays a key role in regulating AMPAR synaptic content during glutamatergic synaptic plasticity^13,17,19,20^. AMPARs constantly switch on the neuronal surface between mobile and immobile states driven by thermal agitation and reversible binding to stable elements such as scaffold or cytoskeletal anchoring slots or extracellular anchors. Even in synapses, AMPARs are not totally stable with around 50% of them moving constantly by Brownian diffusion within the plasma membrane, promoting continuous exchanges between synaptic and extrasynaptic sites^13^. This process is highly regulated by neuronal activity and other stimuli. The majority of AMPARs incorporated into synapses during LTP is from surface diffusion while exocytosed receptors likely serve to replenish the extrasynaptic pool available for subsequent bouts of plasticity^17,20^. This AMPAR redistribution followed by immobilization and accumulation of AMPARs at synapses is the crucial step for the enhanced synaptic transmission during synaptic potentiation^17,20,28,41^. In the present study, we found that HD- causing mutation leads to deregulated AMPAR surface diffusion and that AMPARs fail to stabilize at the surface after cLTP stimuli using complementary HD models including ectopic expression of WT or polyQ–exon1 and of full-length HTT constructs containing WT or polyQ repeats in rat primary neurons. To further study the impact of polyQ expansion on AMPAR mobility, we used neurons from *R6/1* transgenic mice that express the first exon of human HTT with approximately 115 glutamines. These mice, in contrast to the highly used R6/2 model, show reduced symptoms and may represent a less severe form of the polyQ-induced pathology^42^. Since this transgenic model is based on the overexpression of exon1, we also investigated receptor mobility in neurons from two other HD KI mouse models. We first used neurons from *Hdh*^Q111/Q111^ KI mice that are generated on a CD1 background and express a chimeric human HTT exon1 sequence with 111 repeats of CAG in the mouse HTT protein as described previously^43^. We also confirmed the defect in AMPAR mobility using mouse neurons from *Hdh*^*CAG140*/+^ heterozygous KI mice. These mice are generated on a C57/BL6J background and express human HTT exon1 sequence with 140 repeats of CAG as described previously^44^. We chose to use neurons from *Hdh*^*CAG140*/+^ mice rather than BAC models of HD as they recapitulate the genetic situation observed in HD patients (endogenous expression and heterozygous status). In addition, *Hdh*^Q111/Q111^ and *Hdh*^*CAG140*/+^ mice are derived from different genetic constructs. Importantly, *Hdh*^Q111/Q111^ and *Hdh*^*CAG140*/+^ mice show mild phenotypes as compared to *R6/1* mice, further supporting the relevance of the results obtained on AMPAR mobility.

Our findings open a new perspective into the molecular mechanism underlying the impaired hippocampal synaptic plasticity in HD. It is noteworthy that disturbed AMPAR trafficking is also proposed to be one of the first manifestations of synaptic dysfunction that underlies Alzheimer’s disease, which shares many clinical and pathological similarities with HD, such as early-onset cognitive deficiency before perceptible neuronal degeneration. Together with our previous finding that deregulated AMPAR surface diffusion underlies impaired LTP in stress/depression models^21^, these lines of evidence indicate that dysregulation of AMPAR surface diffusion may represent a common molecular basis for the impaired hippocampal synaptic plasticity and memory in various neuronal disorders.

It is generally accepted that BDNF via interaction with TrkB receptors enhances synaptic transmission and plasticity in adult synapses, while its binding to p75^NTR^ has been demonstrated to negatively modulate synaptic plasticity, spine–dendrite morphology, and complexity^11^. Recent studies show that impaired BDNF delivery, as well as the abnormally reduced expression of TrkB receptor and enhanced p75^NTR^ expression account for the hippocampal synaptic and memory dysfunction^12,45,46^. The phosphorylation of GluA1 on Ser-831 through activation of protein kinase C and CaMKII via TrkB receptors has been proposed to be responsible for AMPAR synaptic delivery^11^. However, other evidence indicates that GluA1 phosphorylation at Ser-831 alters single-channel conductance rather than receptor anchoring^47^. Here we provide the first evidence in HD models that administration of BDNF slows down the increased AMPAR surface diffusion possibly via interaction between TARP and PDZ-domain scaffold protein, which is downstream of TrkB-CaMKII signaling pathway. It is possible that both processes, that is, change in the single-channel conductance and receptor anchoring (this study), occur in parallel and affect AMPAR signaling. Interestingly, the reduced CaMKII activity reported in *Hdh*^Q111/Q111^ KI mice could be prevented by normalization of p75^NTR^ levels^12^. This effect could be attributable to the preservation of TrkB signaling, as it has been shown that decreasing p75^NTR^ expression or blocking its coupling to the small GTPase RhoA normalizes TrkB signaling, while upregulation of p75^NTR^ signaling through PTEN (phosphatase and tensin homolog deleted on chromosome 10) results in impaired TrkB signaling^46^. Thus impaired BDNF delivery and aberrant processing of BDNF signal may converge on the TrkB-CaMKII signaling pathway, which impacts the interaction between TARP and PDZ-domain containing scaffold proteins and thus affects AMPAR surface diffusion.

However, the fact that the stargazin–PSD95 interaction is dramatically impaired while the decrease in phosphorylated CaMKII in HD models is modest^12^, indicates that additional signaling pathways downstream or in parallel to BDNF signaling may also contribute to the disruption of stargazin–PSD95 interaction and AMPAR surface trafficking. An imbalance between synaptic versus extrasynaptic *N*-methyl-d-aspartate–type glutamate receptor (NMDAR) activity^48^ has been reported in HD models. NMDAR is known to activate CaMKII, phosphatidylinositol-3 kinase/Akt and the mitogen-activated protein kinase (MAPK) and crosstalk with BDNF signaling pathways. It is possible that multiple signaling pathways synergistically regulate AMPAR surface trafficking.

Although we specifically studied the role of stargazin–PSD95 interaction in regulating AMPAR surface diffusion in HD models, other TARP family members may also be involved. TARP family members (γ-2, γ-3, γ-4, and γ-8) share the binding motif (TTPV) to PDZ-domain scaffold proteins. Notably, γ-8 is the most abundant TARP in the hippocampus^49^. In addition to PSD95, the C-tail of TARPs also enables its potential interaction with other PDZ scaffold proteins, such as SAP97 and SAP102. However, compared to PSD95, SAP97 and SAP102 have much less effect on AMPAR excitatory postsynaptic current^50^. We thus propose that the interaction between TARP and PSD95 may^12^ represent a prominent target in regulating trapping and stabilization of diffusing AMPARs on the postsynaptic membrane.

Tianeptine is a well-tolerated antidepressant primarily used in the treatment of major depressive disorders^51^. It is structurally similar to a tricyclic antidepressant (TCA) but has different pharmacological properties than typical TCAs as it produces its antidepressant effects likely through the alteration of glutamate receptor activity^22,51^. Tianeptine alters glutamatergic transmission, increasing, for instance, the phosphorylation of GluA1 subunits^52^ and activating CaMKII and protein kinase A via the p38, p42/44 MAPK, and c-Jun N-terminal kinase pathways^53^. Through unknown mechanisms, tianeptine prevents stress-induced dendritic atrophy, improves neurogenesis, reduces apoptosis, and normalizes metabolite levels and hippocampal volume^51^. In the present study, we show in complementary HD models that tianeptine restored AMPAR surface diffusion, via BDNF-TrKB signaling pathway, and rescued defective LTP and hippocampal-dependent memory. Interestingly, the activation of BDNF-TrkB signaling pathway is also required for the effect on the depression-like behavior of some typical antidepressants, such as fluoxetine and imipramine^54,55^. The mechanisms underlying chronic tianeptine treatment may involve BDNF-induced neurogenesis^51^; however, our finding that a single-dose administration of tianeptine is sufficient to rescue aberrant LTP in *Hdh*^Q111/Q111^ KI mice points to additional mechanisms. Given the critical role of AMPAR surface diffusion in hippocampal synaptic plasticity, we argue that the beneficial effects of tianeptine on the impaired LTP and hippocampal-dependent memory stem, at least in part, from its normalization of AMPAR surface diffusion. Although tianeptine is also able to augment BDNF intracellular trafficking in WT controls (Supplementary Fig. 4) and immobilize AMPAR surface diffusion under basal conditions^22^, it did not significantly improve hippocampal-dependent memory in WT mice, suggesting that the maintenance of a physiological dynamic equilibrium is key to an effective treatment. The present study also shows beneficial effect of tianeptine on the anxiety/depression-like behavior in *CAG140* KI mouse model. Note that cognitive dysfunction and psychiatric pathologies such as depression, stress, and anxiety are typical features of HD, which occur well before the onset of motor dysfunction, thus the use of tianeptine may represent a promising early therapeutic strategy for HD targeting both psychiatric and cognitive defects. Moreover, that tianeptine is a clinically used drug will facilitate clinical trials.

In conclusion, we unravel AMPAR surface diffusion as a potential novel therapeutic target for early intervention in HD and propose a new therapeutic strategy for HD using the antidepressant tianeptine, which improves hippocampal synaptic and memory deficits as well as anxiety/depression-like behavior in HD mice possibly through the modulation of BDNF signaling and AMPAR surface diffusion.

## Methods

### Transgenic mice, primary neuronal cultures, and transfection

The heterozygous male *R6/1* mice (Jackson Laboratory, Bar Harbor, ME) were crossed with female C57BL/6 mice (Charles River, Lyon). Homozygous *Hdh*^Q111/Q111^ KI mice of HD on CD1 background are generous gift from M.E. MacDonald^56^. The *CAG140* are heterozygous mice with C57Bl6N/J background. The animals were housed with food and water ad libitum under a 12 h light–dark cycle. All work involving animals was conducted according to the rules of ethics of the Committee of University of Bordeaux and the Aquitaine (France, A50120127) and the Institutional Animal Care and Use Committee (European Directive, 2010/63/EU for the protection of laboratory animals, permissions # 92-256B, authorization ethical committee CEEA 26 2012_100). Polymerase chain reaction genotyping with DNA extracted from a piece of tail was carried to identify mice genotype.

Primary cultures of hippocampal neurons were prepared following a previously described method from (1) Sprague-Dawley rats at E18; (2) *Hdh*^Q111/Q111^ KI mice and WT littermates at P0 for AMPAR surface tracking and *Hdh*^Q111/Q111^ KI mice and WT mice at E15 for BDNF intracellular tracking; and (3) *R6*/1 mice and WT littermates at P0^57,58^. Cells were plated at a density of 200 × 10^3^ cells for rat culture and 450 × 10^3^ cells for mice culture per 60 mm dish on poly-lysine pre-coated cover slips. Cultures were maintained in serum-free neurobasal medium (Invitrogen) and kept at 37 °C in 5% CO_2_ for 20 days in vitro (DIV) at maximum. Cells were transfected with appropriate plasmids using Effectene (Qiagen).

### Plasmids and chemical products

Homer 1C::GFP with CaMKII promoter was generated by subcloning homer 1C cDNA into the eukaryotic expression vector pcDNA3 (Invitrogen); EGFP was inserted at the N-terminus of the Homer 1C sequence. Exon1 mutant huntingtin contains 69 polyglutamine expansion (exon1-polyQ-HTT) and WT huntingtin with 17 polyglutamine (exon1-wHTT)^24^. Full-length HTT plasmids encode full-length huntingtin with 17 polyQ (FL-wHTT) or 75Q (FL-polyQ-HTT). GFP fused 480-17Q, 480-68Q huntingtin plasmids encode the first 480 amino acids fragment of huntingtin with 17 (Nter-wHTT) or 68 glutamines (Nter-polyQ-HTT)^59,60^. Tianeptine was purchased from T & W group and MedChemexpress CO., Ltd; BDNF from Sigma-Aldrich; TrkB-Fc from R&D Systems; and kn93 from Tocris. Homemade CB and CB synthesized by Bio S&T were used.

### CB synthesis

CB was synthesized at a 0.05 mmol scale. Amino acids were assembled by automated microwave solid phase peptide synthesis on a CEM microwave–assisted Liberty-1 synthesizer following the standard coupling protocols provided by the manufacturer. Methionine was replaced by Norleucine (λ), a more stable isostere. Linear peptide was cleaved (TFA:H_2_O:EDT:TIS, 94:2.5:2.5:1) and purified by high-performance liquid chromatography (HPLC). Disulfide bond formation was carried out for 10 h in H_2_0 in the presence of dimethyl sulfoxide (5%) and ammonium acetate (0.05 M) at high dilution of the peptide (100 µM). Solvent excess was removed and the peptide was purified by reversed-phase (RP)-HPLC (YMC C18, ODS-A 5/120, 250 × 20 mm^2^, ultraviolet detection at 228 and 280 nm, using a standard gradient: 5% MeCN containing 0.1% TFA for 5 min followed by a gradient from 10% to 40% over 40 min in dH_2_O containing 0.1% trifluoroacetic acid (TFA) at a flow rate of 12 ml min^−1^). Peptides were characterized by analytic RP-HPLC and matrix-assisted light desorption/ionization. Peptides were quantified by absorbance measurement at 280 nm, aliquoted, lyophilized, and stored at −80 °C until usage.

### Single-particle tracking of AMPAR using QD

Rat primary hippocampal neurons were co-transfected at DIV 10–11 with GFP/homer1c-GFP and wHTT/polyQ-HTT at the ratio of 1:9 to ensure that the majority of GFP-transfected neurons were transfected with HTT. Homer1c was used as a postsynaptic marker. Endogenous GluA2 and GluA1 QD tracking was performed at DIV 11–12 as previously described^22^. Neurons were first incubated with mouse monoclonal antibody against N-terminal extracellular domain GluA2 subunit (a kind gift from E. Gouaux, Oregon Health and Science University, USA) (1:1000) or rabbit polyclonal antibody against N-terminal extracellular domain GluA1 subunit (PC246, Calbiochem) (1:200) for 7 min, followed by incubation with QD 655 Goat F(ab’)2 anti-mouse (Invitrogen, 1:10,000 to 1:20,000) or anti-Rabbit IgG (Invitrogen, 1:10,000 to 1:20,000) for 4 min. The specificity of the antibodies was controlled using hippocampal neuron culture from GluA2- or GluA1-knockout mice in our laboratory (data not shown). Non-specific binding was blocked by 5% bovine serum albumin (Sigma-Aldrich). QDs were detected by using a mercury lamp and appropriate excitation/emission filters. Images were obtained with an interval of 50 ms and up to 1000 consecutive frames. Signals were detected using a CCD camera (Quantem, Roper Scientific). QDs were followed on randomly selected dendritic regions for up to 20 min. QD recording sessions were processed with the MetaMorph software (Molecular Devices, Sunnyvale, USA). The instantaneous diffusion coefficient, *D*, was calculated for each trajectory, from linear fits of the first 4 points of the mean-square-displacement versus time function using MSD(*t*) = < *r*^2^ > (*t*) = 4*Dt*. The two-dimensional trajectories of single molecules in the plane of focus were constructed by correlation analysis between consecutive images using a Vogel algorithm. QD-based trajectories were considered synaptic if colocalized with Homer 1C dendritic clusters for at least five frames.

### BDNF intracellular transport

Rat hippocampal neurons were co-transfected at DIV 9–10 with GFP-fused 480-17Q (GFP::Nter-wHTT) or 480-68Q (GFP::Nter-polyQ-HTT) and mCherry-BDNF at the ratio of 4:1 using Effectene (QIAGEN). Live imaging was carried out at DIV 10–11. The movement of BDNF-containing vesicles was tracked using video microscopy on an inverted Leica DMI 6000 Year microscope (Leica Microsystems, Wetzlar, Germany) equipped with a HQ2 camera (Photometrics, Tucson, USA). The objective HCX PL used was a CS APO ×63 NA 1.32 oil. The atmosphere was 37 °C incubator created with year box and air heating system (Life Imaging Services, Basel, Switzerland). Acquisitions and calculation were done on the MetaMorph software (Molecular Devices, Sunnyvale, USA). For BDNF axonal trafficking in hippocampal neurons of mouse *Hdh*^Q111/Q111^ KI and WT mice, hippocampal neurons at E15 were used. Microchambers, neuronal transfection as well as videomicroscopy were previously described^27^. Briefly, images were collected in stream mode using a Micromax camera (Roper Scientific) with an exposure time of 100–150 ms. Projections, animations, and analyses were generated using the ImageJ software (http://rsb.info.nih.gov/ij/, NIH, USA). Maximal projection was performed to identify the vesicle paths, which in our system corresponds to vesicle movements in axons. Kymographs and analyses were generated with the KymoToolBox, a home-made plug-in^27^.

### Super-resolution imaging of AMPAR surface diffusion

uPAINT was used. Rat primary hippocampal neurons were co-transfected at DIV 4 with homer1c-GFP and FL-wHTT/polyQ-HTT for 2 weeks. Homer1c was used as a postsynaptic marker. Atto647-conjugated mouse monoclonal antibody against N-terminal extracellular domain GluA2 subunit (a kind gift from E. Gouaux, Oregon Health and Science University, USA) (1:5000–1:10,000) was used. The microscope was a Nikon Ti Eclipse (Nikon France S.A.S., Champigny-sur-Marne, France) equipped with a the Perfect Focus System (PFS) and a TIRF arm and using objective Apo TIRF ×100 oil NA 1.49 and a sensitive Evolve EMCCD camera (Photometrics, Tucson, USA). The diode lasers used were at 491 and 635 nm. This system is equipped by a motorized stage Ti-S-ER. The 37 °C atmosphere was created with an incubator box and an air heating system (Life Imaging Services, Basel, Switzerland). This system was controlled by the MetaMorph software (Molecular Devices, Sunnyvale, USA).

### BDNF enzyme-linked immunosorbent assay

The BDNF concentration was evaluated using the BDNF ELISA Kit (Millipore, Abnova) following the protocol provided by the company.

### Western blot

Western blot is performed as previously described^22^. Ten µg of protein was loaded per lane and analyzed by sodium dodecyl sulfate-polyacrylamide gel electrophoresis (SDS–PAGE). Primary antibodies anti-BDNF antibody (Santa Cruz Biotechnology, sc-546, 1:200) and anti-Tubulin antibody (Sigma-Aldrich, T5168, 1:4000) were used.

### Co-immunoprecipitation

For each experiment, one mouse brain of 5-week-old littermate WT or HDH-Q111 were homogenized in 6 ml of homogenization buffer (20 mM Hepes, 0.15 mM EDTA, 0.4 mM EGTA, 10 mM KCl, pH 7.5, cocktail of protease inhibitors (aprotinin, leupeptin, pepstatin-A, MG132, and pefabloc, 10 µg/ml) and centrifuged for 10 min at 860 × *g*. The supernatant was centrifuged for 30 min at 17,000 × *g*. The pellet was homogenized with 20 strokes in 15 ml of the same buffer adjusted at 15% sucrose. The homogenate was centrifuged for 10 min at 860 × *g*, in order to remove genomic DNA. The supernatant containing the membranes was centrifuged again for 30 min at 17,000 × *g*. Brain membranes were solubilized in a medium containing 20 mM Hepes, 1% Triton-X100, 150 mM NaCl, 0.15 mM EDTA, 4 mM EGTA (pH 7.5) and the antiprotease cocktail, with 20 dunces with a potter. Sample was centrifuged for 45 min at 17,000 × *g*. All steps were performed at 4 °C. Protein concentration was evaluated with a BCA assay. Triton-X100 supernatant (0.5 mg) was incubated with the anti-stargazin antibody (AB-9876, Millipore, 1:150) for 1 h at 4 °C and then incubated overnight with 50 µl of protein-A Sepharose at 4 °C. Resin was washed with 1 ml of loading buffer and 0.5 ml of the same buffer containing 500 mM NaCl. Beads were resuspended in 100 µl of gel loading buffer, run on 4–15% SDS-PAGE, and immunoblotted with anti-stargazin (AB-9876, Millipore, 1:1000) and anti-PSD95 (Neuromab, 75-028, 1:1000) antibodies. Pictures and quantitative analysis of western blots were performed using the Li-Cor apparatus (Odyssey scanner v3.0).

### DUOLINK in situ PLA

PLA was performed to evaluate the interaction between stargazin and PSD95 in situ using Duolink In Situ Detection Reagents (Sigma DUO92013) following the following protocol: https://www.sigmaaldrich.com/technical-documents/protocols/biology/duolink-fluorescence-user-manual.html. Antibodies were anti-stargazin (Millipore 07-577, 1:100) and anti-PSD95 (Thermo Scientific MA1-046, 1:100).

### Extracellular recording in hippocampal CA1 pyramidal neurons

Male heterozygous *R6/1* mice, *Hdh*^Q111/Q111^ KI mice and respective WT littermates were used for ex vivo extracellular recording. *Hdh*^Q111/Q111^ KI mice (10–12 weeks of age) received a single injection of tianeptine (i.p., 10 mg kg^−1^) with glucose as negative control; *R6/1* mice received chronic tianeptine treatment (25 mg kg^−1^, i.p. daily) starting from 4 weeks of age until 12 weeks of age. As described previously^22^, a hippocampal slice was transferred to a superfusing recording chamber with temperature controlled at 33.5 °C and continuously perfused with oxygenated artificial cerebrospinal fluid (ACSF) using a peristaltic pump (Ismatec, Switzerland). A teflon-coated tungsten bipolar stimulating electrode (Phymep, Paris, France) was positioned in stratum radiatum, allowing the afferent Schaffer collateral–commissural pathway from the CA3 area to the CA1 region to be stimulated. The fEPSPs were recorded from stratum radiatum of CA1 area, using a glass electrode (3–5 MΩ) pulled from borosilicate glass tubing (Harvard Apparatus, USA; 1.5 mm O.D x 1.17 mm I.D) and filled with ACSF. Pulses were delivered at 7.5 s by a stimulus isolator (Isoflex, AMPI, Jerusalem, Israel), with adjusting current intensity to obtain 30–40% of the maximum fEPSP. A theta-burst stimulation (TBS) protocol (4 pulses, respectively, delivered at 100 Hz, repeated 10 times, at an interval of 200 ms) was delivered by Clampex10.4 (Molecular Devices, USA) and the stimulus isolator to induce LTP. Recordings were made continually for >60 min, following the TBS. Data were recorded with a Multiclamp700B (Axon Instruments, USA) and acquired with Clampex10.4. The slope of the fEPSP was measured using the clampfit10.4 software, with all values normalized to a 5 min baseline period; the values of potentiation during 50–60 min after TBS were reported in the figures. The percentages of potentiation during the last 10 min of each recordings were plotted in cumulative probability curves. We did not include recordings in which the fiber volley changed >30% from the baseline value or the global evolution. No difference in fiber volley time course was detected between the compared groups. The recording was performed blindly in terms of mice genotype and treatment.

### Behavioral tests

Male *R6/1* and WT littermate mice were used for behavioral tests. At 4 weeks of age, littermate mice with mixed genotypes were housed (3–5 per cage) in polycarbonate standard cages (33 × 15 × 14 cm^3^) and randomly allocated to vehicle or drug treatment groups. Simple randomization method was used for allocating WT and *R6/1* mice to vehicle or drug treatment groups. The sample size was chosen based on previous experience and publications. Mice received daily i.p. injection of 0.9% saline (vehicle) or tianeptine (10 mg kg^−1^) dissolved in 0.9% saline until 12 weeks of age, when the animals were subjected to a battery of behavioral tests. On day 1, all mice were subjected to open field test; on day 2, spatial memory was assessed in Y-maze. Following a week of rest, on day 9, a subset of mice was further tested for contextual fear conditioning, which is performed lastly in order to minimize confounding factors. All behavioral testing was carried out in the light phase (light intensity: 45–50 lux). Before each behavioral test, mice were individually housed in standard cages with sawdust, food, and water and left undisturbed in the experimental room at least 30 min before testing began. Behavioral tests were performed blindly concerning mice genotype and treatment.

### Open field

The apparatus constituted of a white square arena (42 × 42 × 20 cm^3^). Each animal was placed in the center of the arena and allowed to explore for 20 min. Images tracked from a camera above the maze were analyzed with Ethovision (version 9.1). The total distance traveled and the time spent moving were analyzed as readouts of locomotor activity. The apparatus was cleaned by ethanol 70% between mice.

### Y-maze

Hippocampal-dependent spatial working memory was evaluated using Y-maze. The apparatus consisted of three identical gray plastic arms (42 × 8 × 15 cm^3^) and spaced at 120° of each other. The maze was located in the middle of a room containing a variety of extramaze cues. A digital camera was mounted above the maze transmitting the data to a PC running the Ethovision system. Mice were assigned two arms (start and familiar arm) to which they were exposed during the first phase of the test (habituation phase). The remaining third arm blocked by a gray plastic door constituted the novel arm during the second phase (testing phase). Mice were placed at the end of the start arm and allowed to explore freely both the start and the other unblocked arm for 5 min before being removed from the maze and returned to the waiting cage. After 10 min in the waiting cage, the test phase began. During this phase, the door was removed and all three arms were unblocked; mice were placed at the end of the start arm and allowed to explore the entire maze for 2 min. During the habituation and testing phase, video timer was started once the mouse had left the start arm and entered the center, thus, two mice which did not leave start arm during test phase were excluded from the analysis. The apparatus was cleaned between the two phases in order to avoid olfactory cues. Time spent in the novel arm in comparison to time in all three arms was used as one readout for hippocampal-dependent spatial memory. Y-maze analysis was carried out blindly with respect to genotype or treatment.

### Contextual fear conditioning

Contextual fear conditioning provides a measure of memory by assessing a memory for the association between mild foot shock and a salient environmental cue. In the fear conditioning test, freezing behavior is defined as the complete lack of movement, which is a characteristic fear response in rodents, providing a readout of hippocampal-dependent memory. Fear conditioning was performed in a testing chamber with internal dimensions of 25 × 25 × 25 cm^3^, which has transparent plastic walls each side and steel bars on the floor. A camera mounted at one side recorded each session. The chamber was located inside a larger, insulated, transparent plastic cabinet (67 × 53 × 55 cm^3^) that provided protection from outside noise. The cabinet contained a ventilation fan that was operated during the sessions. Mice were held outside the experimental room in individual cages prior to testing. Training chambers were cleaned with 100% ethanol solution before and after each trial to avoid any olfactory cues. The experiments ran over 2 consecutive days. On Day 1, mice were placed in the conditioning chamber and 2 min 28 s later received one footstock (2 s, 0.3 mA). Mice were removed from the chamber 30 s after the shock. On Day 2, they returned to the same conditioning chamber for a 3-min period in the exact same conditions as Day 1 but without electrical shock, to evaluate context-induced freezing. Contextual fear conditioning experiment was carried out blindly with respect to genotype or treatment.

### Elevated plus maze (EPM)

Male *CAG 140* heterozygous KI mice received daily i.p. injection of saline or tianeptine at 10 mg kg^−^^1^ at 12 weeks of age. No randomization methods were used for allocating WT and *CAG 140* mice to vehicle or drug treatment groups. Behavioral tests were performed at 16 weeks of age unblindly. Each animal, over a week, was successively tested in the EPM and NSF, which represent different anxiety and depression behavior paradigms. Behavioral tests were performed during the light phase between 0700 and 1900 hours. EPM was performed as previously^61^. The maze is a plus-cross-shaped apparatus, with two open arms and two arms closed by walls linked by a central platform 50 cm above the floor. Mice were individually put in the center of the maze facing an open arm and were allowed to explore the maze during 5 min. The time spent in and the number of entries into the open arms were used as an anxiety index. Locomotion was also measured to ensure any confounding effects. All parameters were measured using a videotracker (EPM3C, Bioseb, Vitrolles, France).

### Novelty suppressed feeding (NSF)

The NSF is a conflict test that elicits competing motivations: the drive to eat and the fear of venturing into the center of a brightly lit arena. The latency to begin to eat is used as an index of anxiety/depression-like behavior, because classical anxiolytic drugs as well as chronic antidepressants decrease this measure. The NSF test was carried out during a 15-min period as previously described^61^. Briefly, the testing apparatus consisted of a plastic box (50 × 50 × 20 cm^3^), the floor of which was covered with approximately 2 cm of wooden bedding. Twenty-four hours prior to behavioral testing, all food was removed from the home cage. At the time of testing, a single pellet of food (regular chow) was placed on a white paper platform positioned in the center of the box. Each animal was placed in a corner of the box, and a stopwatch was immediately started. The latency to eat (defined as the mouse sitting on its haunches and biting the pellet with the use of forepaws) was timed. Immediately afterwards, the animal was transferred to its home cage, and the amount of food consumed by the mouse in the subsequent 5 min was measured, serving as a control for change in appetite as a possible confounding factor.

### Statistics

Statistical analysis was performed using Prism 7.04 (GraphPad, USA). D’Agostino & Pearson normality test and Shapiro–Wilk normality test were used to test normal distribution. For two-group comparison, *F* test was used to test variance within each group; for multiple group comparison, Brown–Forsythe test and Bartlett’s test were used. Datasets, which were not normally distributed or with unequal population variances, were transformed by converting the values to their logarithms or square roots to fit Gaussian distribution or to equalize the standard deviations. Data values were shown as mean ± s.e.m. for normally distributed data; median ± 95% confidence interval (95% c.i.) or median ± interquartile range for non-normally distributed data. Normally distributed datasets with equal population variances were compared using paired or unpaired Student’s *t* test (for two-group, one-factor comparison), one-way analysis of variance (ANOVA) test followed by Tukey’s multiple comparison test (for multi-group, one-factor comparison), or two-way ANOVA followed by Tukey’s multiple comparison test (for multi-group, two-factor comparison). Non-normally distributed datasets were tested by non-parametric Mann–Whitney test (for two-group, one-factor comparison) or Kruskal–Wallis test followed by Dunn’s multiple comparison test (for multi-group, one-factor comparison). Cumulative probability curves were compared using Mann–Whitney/Wilcoxon’s test. Statistical significance was indicated by *P* values: *P < 0.05, **P < 0.01, and ***P < 0.001.

## Electronic supplementary material

Supplementary Information

## Data Availability

The authors declare that all the data supporting the findings of this study are available from the authors on reasonable request.

## References

[1] Berrios GE (2002). Psychiatric symptoms in neurologically asymptomatic Huntington’s disease gene carriers: a comparison with gene negative at risk subjects. Acta Psychiatr. Scand..

[2] Tabrizi SJ (2011). Biological and clinical changes in premanifest and early stage Huntington’s disease in the TRACK-HD study: the 12-month longitudinal analysis. Lancet Neurol..

[3] Saudou F, Humbert S (2016). The biology of huntingtin. Neuron.

[4] Giralt A (2009). Brain-derived neurotrophic factor modulates the severity of cognitive alterations induced by mutant huntingtin: involvement of phospholipaseCgamma activity and glutamate receptor expression. Neuroscience.

[5] Hodgson JG (1999). A YAC mouse model for Huntington’s disease with full-length mutant huntingtin, cytoplasmic toxicity, and selective striatal neurodegeneration. Neuron.

[6] Lynch G (2007). Brain-derived neurotrophic factor restores synaptic plasticity in a knock-in mouse model of Huntington’s disease. J. Neurosci..

[7] Murphy KP (2000). Abnormal synaptic plasticity and impaired spatial cognition in mice transgenic for exon 1 of the human Huntington’s disease mutation. J. Neurosci..

[8] Milnerwood AJ (2006). Early development of aberrant synaptic plasticity in a mouse model of Huntington’s disease. Hum. Mol. Genet..

[9] Chan AW (2014). A two years longitudinal study of a transgenic Huntington disease monkey. BMC Neurosci..

[10] Majerova V (2012). Disturbance of real space navigation in moderately advanced but not in early Huntington’s disease. J. Neurol. Sci..

[11] Park H, Poo MM (2013). Neurotrophin regulation of neural circuit development and function. Nat. Rev. Neurosci..

[12] Brito V (2014). Neurotrophin receptor p75(NTR) mediates Huntington’s disease-associated synaptic and memory dysfunction. J. Clin. Invest..

[13] Choquet D, Triller A (2013). The dynamic synapse. Neuron.

[14] Shepherd JD, Huganir RL (2007). The cell biology of synaptic plasticity: AMPA receptor trafficking. Annu. Rev. Cell Dev. Biol..

[15] Volk L, Chiu SL, Sharma K, Huganir RL (2015). Glutamate synapses in human cognitive disorders. Annu. Rev. Neurosci..

[16] Kessels HW, Malinow R (2009). Synaptic AMPA receptor plasticity and behavior. Neuron.

[17] Penn AC (2017). Hippocampal LTP and contextual learning require surface diffusion of AMPA receptors. Nature.

[18] Huganir RL, Nicoll RA (2013). AMPARs and synaptic plasticity: the last 25 years. Neuron.

[19] Borgdorff AJ, Choquet D (2002). Regulation of AMPA receptor lateral movements. Nature.

[20] Makino H, Malinow R (2009). AMPA receptor incorporation into synapses during LTP: the role of lateral movement and exocytosis. Neuron.

[21] Groc L, Choquet D, Chaouloff F (2008). The stress hormone corticosterone conditions AMPAR surface trafficking and synaptic potentiation. Nat. Neurosci..

[22] Zhang H (2013). Regulation of AMPA receptor surface trafficking and synaptic plasticity by a cognitive enhancer and antidepressant molecule. Mol. Psychiatry.

[23] Pla P, Orvoen S, Saudou F, David DJ, Humbert S (2014). Mood disorders in Huntington’s disease: from behavior to cellular and molecular mechanisms. Front. Behav. Neurosci..

[24] Saudou F, Finkbeiner S, Devys D, Greenberg ME (1998). Huntingtin acts in the nucleus to induce apoptosis but death does not correlate with the formation of intranuclear inclusions. Cell.

[25] Caldeira MV (2007). Brain-derived neurotrophic factor regulates the expression and synaptic delivery of alpha-amino-3-hydroxy-5-methyl-4-isoxazole propionic acid receptor subunits in hippocampal neurons. J. Biol. Chem..

[26] Gauthier LR (2004). Huntingtin controls neurotrophic support and survival of neurons by enhancing BDNF vesicular transport along microtubules. Cell.

[27] Zala D (2013). Vesicular glycolysis provides on-board energy for fast axonal transport. Cell.

[28] Opazo P (2010). CaMKII triggers the diffusional trapping of surface AMPARs through phosphorylation of stargazin. Neuron.

[29] Lee SJ, Escobedo-Lozoya Y, Szatmari EM, Yasuda R (2009). Activation of CaMKII in single dendritic spines during long-term potentiation. Nature.

[30] Massa SM (2010). Small molecule BDNF mimetics activate TrkB signaling and prevent neuronal degeneration in rodents. J. Clin. Invest..

[31] Nitta A (1999). 4-methylcatechol increases brain-derived neurotrophic factor content and mRNA expression in cultured brain cells and in rat brain in vivo. J. Pharmacol. Exp. Ther..

[32] Simmons DA (2009). Up-regulating BDNF with an ampakine rescues synaptic plasticity and memory in Huntington’s disease knockin mice. Proc. Natl. Acad. Sci. USA.

[33] Reagan LP (2007). Tianeptine increases brain-derived neurotrophic factor expression in the rat amygdala. Eur. J. Pharmacol..

[34] Zoladz PR, Park CR, Munoz C, Fleshner M, Diamond DM (2008). Tianeptine: an antidepressant with memory-protective properties. Curr. Neuropharmacol..

[35] Menalled L (2009). Systematic behavioral evaluation of Huntington’s disease transgenic and knock-in mouse models. Neurobiol. Dis..

[36] Cazorla M (2010). Cyclotraxin-B, the first highly potent and selective TrkB inhibitor, has anxiolytic properties in mice. PLoS ONE.

[37] Lu W (2001). Activation of synaptic NMDA receptors induces membrane insertion of new AMPA receptors and LTP in cultured hippocampal neurons. Neuron.

[38] Constals A (2015). Glutamate-induced AMPA receptor desensitization increases their mobility and modulates short-term plasticity through unbinding from Stargazin. Neuron.

[39] LeDoux JE (2012). Evolution of human emotion: a view through fear. Prog. Brain Res..

[40] Maren S, Aharonov G, Fanselow MS (1997). Neurotoxic lesions of the dorsal hippocampus and Pavlovian fear conditioning in rats. Behav. Brain Res..

[41] Petrini EM (2009). Endocytic trafficking and recycling maintain a pool of mobile surface AMPA receptors required for synaptic potentiation. Neuron.

[42] Mangiarini L (1996). Exon 1 of the HD gene with an expanded CAG repeat is sufficient to cause a progressive neurological phenotype in transgenic mice. Cell.

[43] Wheeler VC (1999). Length-dependent gametic CAG repeat instability in the Huntington’s disease knock-in mouse. Hum. Mol. Genet..

[44] Menalled LB (2002). Early motor dysfunction and striosomal distribution of huntingtin microaggregates in Huntington’s disease knock-in mice. J. Neurosci..

[45] Parsons MP, Raymond LA (2014). It’s not necessarily all about the delivery in Huntington’s disease. Neuron.

[46] Plotkin JL (2014). Impaired TrkB receptor signaling underlies corticostriatal dysfunction in Huntington’s disease. Neuron.

[47] Hayashi Y (2000). Driving AMPA receptors into synapses by LTP and CaMKII: requirement for GluR1 and PDZ domain interaction. Science.

[48] Okamoto S (2009). Balance between synaptic versus extrasynaptic NMDA receptor activity influences inclusions and neurotoxicity of mutant huntingtin. Nat. Med..

[49] Nicoll RA (2003). Expression mechanisms underlying long-term potentiation: a postsynaptic view. Philos. Trans. R. Soc. Lond. B Biol. Sci..

[50] Schnell E (2002). Direct interactions between PSD-95 and stargazin control synaptic AMPA receptor number. Proc. Natl. Acad. Sci. USA.

[51] McEwen BS (2010). The neurobiological properties of tianeptine (Stablon): from monoamine hypothesis to glutamatergic modulation. Mol. Psychiatry.

[52] Svenningsson P (2007). Involvement of AMPA receptor phosphorylation in antidepressant actions with special reference to tianeptine. Eur. J. Neurosci..

[53] Szegedi V (2011). Tianeptine potentiates AMPA receptors by activating CaMKII and PKA via the p38, p42/44 MAPK and JNK pathways. Neurochem. Int..

[54] Monteggia LM (2004). Essential role of brain-derived neurotrophic factor in adult hippocampal function. Proc. Natl. Acad. Sci. USA.

[55] Saarelainen T (2003). Activation of the TrkB neurotrophin receptor is induced by antidepressant drugs and is required for antidepressant-induced behavioral effects. J. Neurosci..

[56] Wheeler VC (2000). Long glutamine tracts cause nuclear localization of a novel form of huntingtin in medium spiny striatal neurons in HdhQ92 and HdhQ111 knock-in mice. Hum. Mol. Genet..

[57] Pineda JR (2009). Genetic and pharmacological inhibition of calcineurin corrects the BDNF transport defect in Huntington’s disease. Mol. Brain.

[58] Zhang H (2013). NGF rescues hippocampal cholinergic neuronal markers, restores neurogenesis, and improves the spatial working memory in a mouse model of Huntington’s Disease. J. Huntingt. Dis..

[59] Humbert S (2002). The IGF-1/Akt pathway is neuroprotective in Huntington’s disease and involves Huntingtin phosphorylation by Akt. Dev. Cell.

[60] Pardo R (2006). Inhibition of calcineurin by FK506 protects against polyglutamine-huntingtin toxicity through an increase of huntingtin phosphorylation at S421. J. Neurosci..

[61] Mendez-David I (2014). Rapid anxiolytic effects of a 5-HT(4) receptor agonist are mediated by a neurogenesis-independent mechanism. Neuropsychopharmacology.

