# Supplementary material for: Modulation of AMPA receptor surface diffusion restores hippocampal plasticity and memory in Huntington’s disease models
Source: Nat Commun. 2018 Oct 15;9:4272. doi: 10.1038/s41467-018-06675-3 (PMC6189172; doi:10.1038/s41467-018-06675-3)
Supplement: Supplementary file 1 — Supplementary Information [file 41467_2018_6675_MOESM1_ESM.pdf]

Modulation of AMPA receptor surface diffusion restores hippocampal plasticity and memory in Huntington's disease models

SUPPLEMENTARY INFORMATION

Zhang et al.

Supplementary Figures

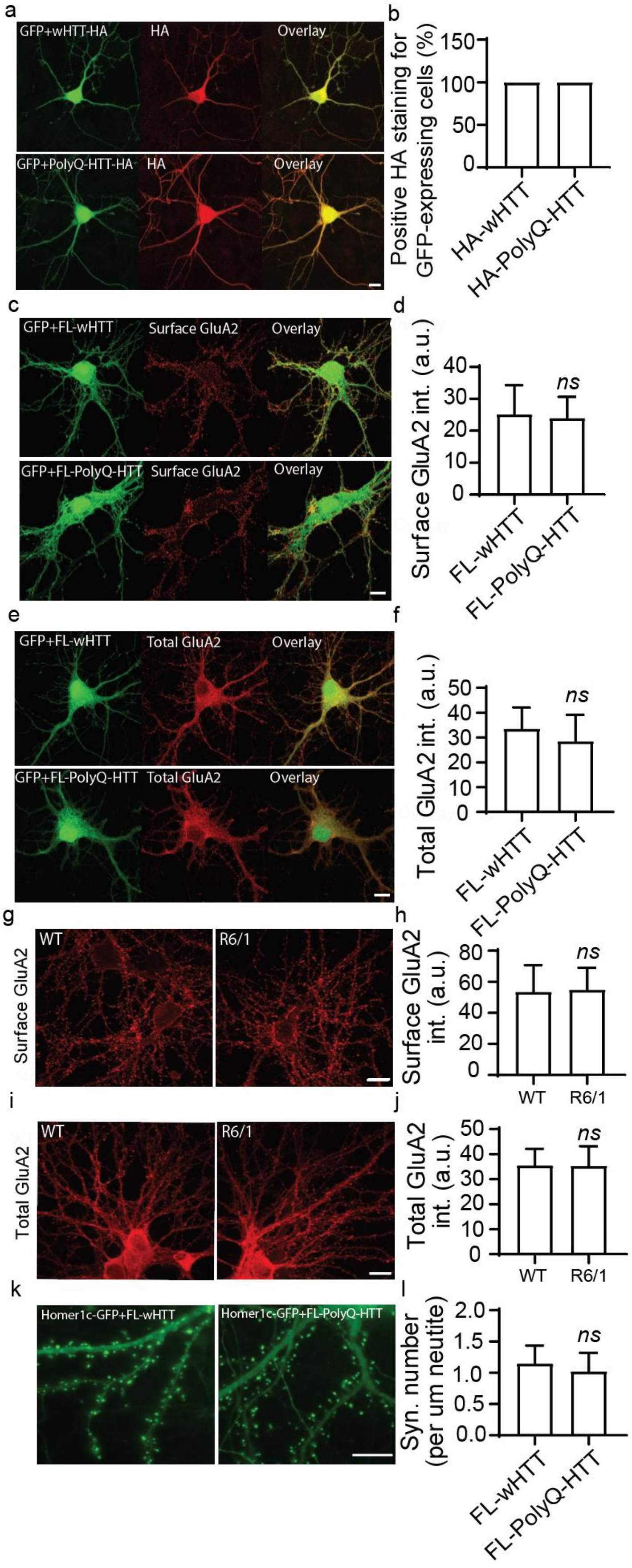

Supplementary Figure 1 Surface and total AMPAR as well as synapse number were not significantly different between WT and HD models (**a, b**) Typical images (**a**) and quantification (**b**) for hippocampal neurons co-transfected with GFP and wHTT or PolyQ-HTT fused with HA at the ratio of 1:4 followed by HA staining. 100% of GFP expressing cells have positive HA staining, n = 30 and 29 cells from 2 separate experiments. (**c, d**) Typical images (**c**) for hippocampal neurons co-transfected with GFP and wHTT or PolyQ-HTT followed by surface GluA2-AMPAR staining. Surface GluA2 intensity (int.) was quantified and shown as arbitrary unit (a.u.) (**d**); n = 17 and 22 cells from 2 separate experiments. (**e, f**) Typical images (**e**) for hippocampal neurons co-transfected with GFP and wHTT or PolyQ-HTT followed by total GluA2-AMPAR staining. Total GluA2 int. was quantified (**f**); n = 19 and 16 cells from 2 separate experiments. (**g, h**) Typical images (**g**) for hippocampal neurons from WT and R6/1 mice with surface GluA2-AMPAR staining. Surface GluA2 int. was quantified (**h**); n = 29 and 29 cells from 2 separate experiments. (**i, j**) Typical images (**i**) for hippocampal neurons from WT and R6/1 mice with total GluA2-AMPAR staining. Total GluA2 int. was quantified (**j**); n = 30 and 30 cells from 2 separate experiments. (**k,l**) Typical images (**k**) from neurons co-transfected with homer1c- GFP and wHTT or PolyQ-HTT. Synapse number was quantified (**l**); n = 13 and 16 cells from 3 separate experiments. Scale bars, 10μm. Data values are mean ± s.e.m (**c-l**); Significance was determined by unpaired two-tailed Student's *t*-test. *ns*, no significance.

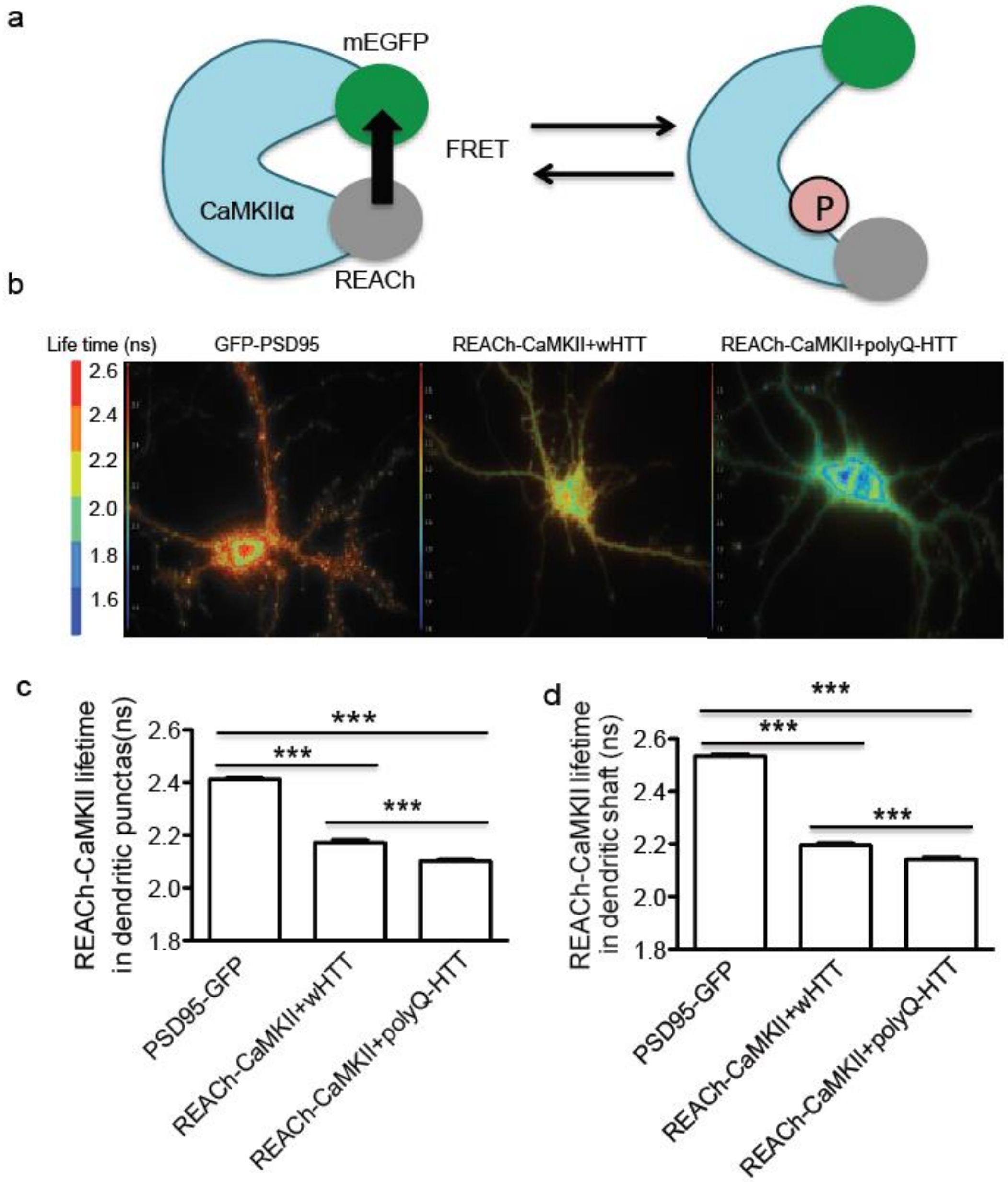

Supplementary Figure 2 Decreased CaMKII activity in HD cellular model (**a**) Schematic diagram showing fluorescence resonance energy transfer (FRET)-based CamKIIα, named REACh-CamKIIα. The activation of REACh-CaMKII changes the conformation to the open state in which its kinase domain is exposed, thereby decreasing FRET and increasing the fluorescence lifetime of mEGFP. (**b**) Representative lifetime image of rat hippocampal neurons expressing PDS95-GFP, REACh-CaMKIIα plus FL-wHTT, and REACh-CaMKIIα plus FL-polyQ-HTT. Blue color indicates strong FRET and short lifetime, while red color represents weak FRET and long lifetime. (**c, d**) Quantification of lifetime in dendritic puncta (**c**) or in dendritic shaft (**d**) in randomly-selected regions of rat hippocampal neurons expressing PDS95-GFP, REACh-CaMKIIα plus FL-wHTT, or REACh-CaMKIIα plus FL-polyQ-HTT. PDS95-GFP-expressing neurons showed long lifetime (≥ 2.4 ns) in both dendritic puncta and shaft indicating no FRET. Lower lifetime indicates stronger FRET and reduced CamKIIα activity; data are mean ± s.e.m; n = 178, 231, and 238 regions for dendritic puncta, and n = 93, 115 and 188 regions for dendritic shaft in neurons expressing PDS95-GFP, REACh-CaMKIIα plus FL-wHTT, and REACh-CaMKIIα plus FL-polyQ-HTT, respectively. Significance was assessed by One-way ANOVA followed by Tukey's multiple comparison test; *** $P < 0.001$.

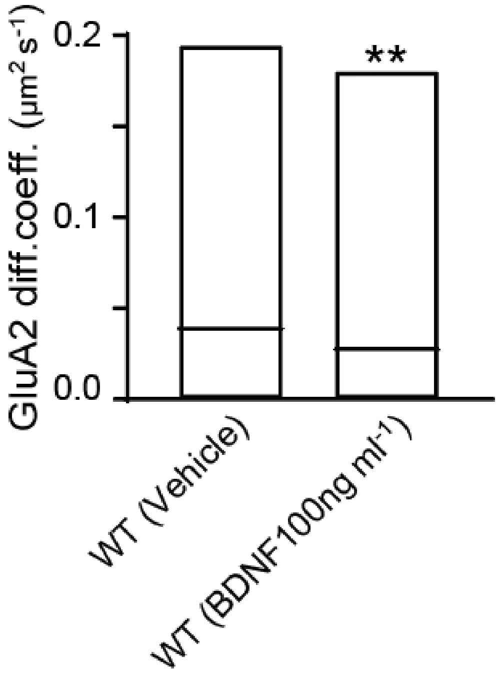

Supplementary Figure 3 GluA2-AMPAR diffusion coefficients (median ± 25-75% interquartile range (IQR)) in WT rat hippocampal neurons treated with vehicle or BDNF; n = 11695 and 16441 trajectories from 14 and 17 cells, respectively. Significance was determined by Mann-Whitney test; $^{**}P < 0.01$.

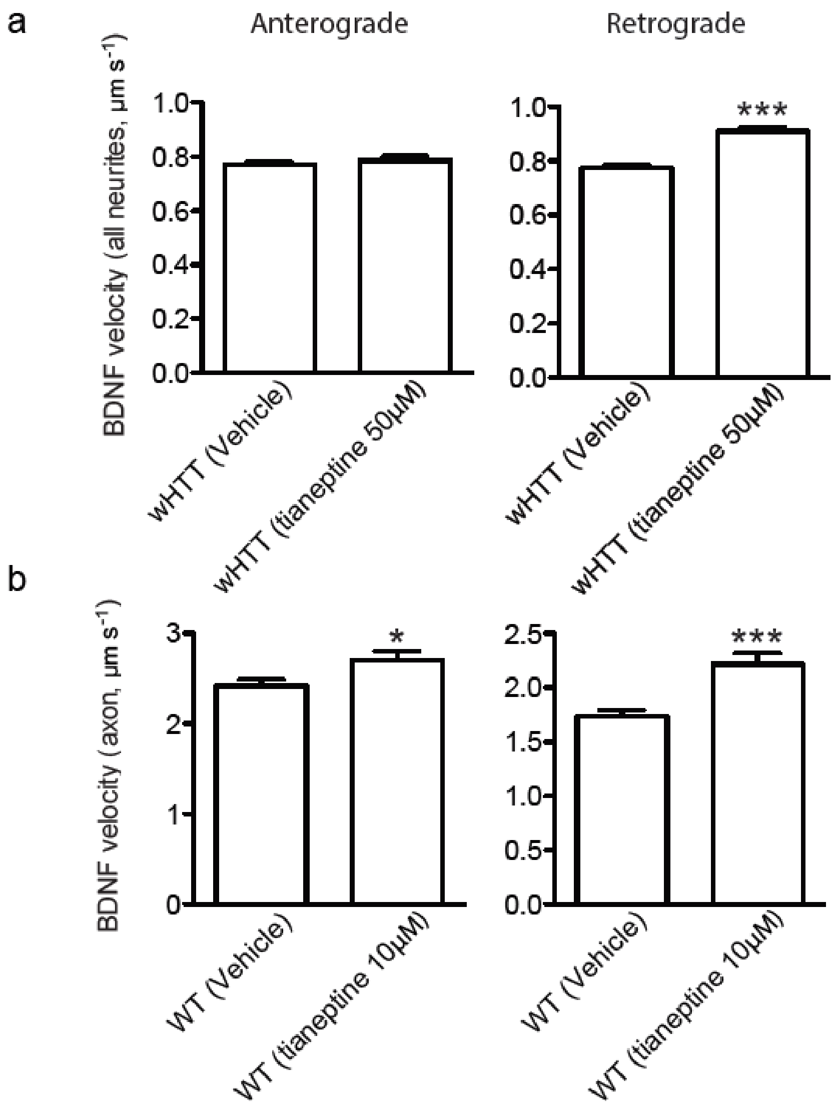

Supplementary Figure 4 Tianeptine facilitated BDNF intracellular transport in wHTT- expressing rat hippocampal neurons and neurons from WT mice. (**a, b**) Anterograde and retrograde BDNF transport velocity in all neurites of vehicle- or tianeptine-treated wHTT- expressing rat hippocampal neurons (**a**), and in the axon of hippocampal neurons from WT and *Hdh*$^{Q111/Q111}$ mice (**b**); values are mean ± s.e.m; n = 5569, 2522, 5227 and 2542 trajectories for anterograde and retrograde BDNF velocity in vehicle and tianeptine-treated wHTT-expressing neurons, respectively; n = 236, 157, 194 and 110 trajectories for anterograde and retrograde BDNF velocity in vehicle and tianeptine-treated neurons from WT and *Hdh*$^{Q111/Q111}$ mice. Significance was determined by unpaired two-tailed Student's *t*-test; *$P < 0.05$, ***$P < 0.001$.

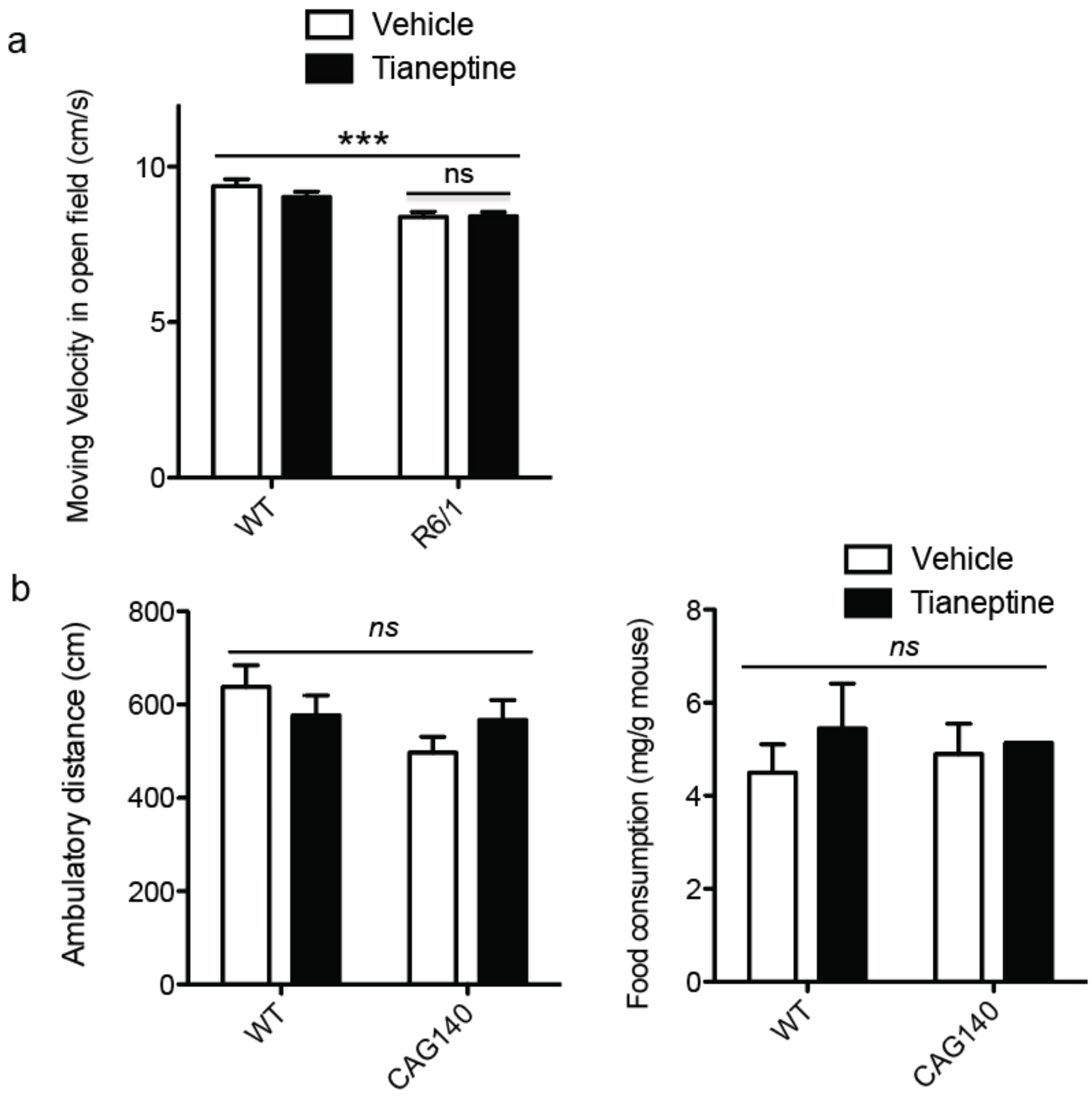

Supplementary Figure 5 Tianeptine did not affect moving velocity of R6/1 mice in Open Field test, either change ambulatory distance or food assumption of CAG140 mice in elevated plus maze (EPM) and novelty-suppressed feeding (NSF) tests, respectively. (**a**) Moving velocity in open field was significantly different between genotypes but not between treatments; values are mean ± s.e.m; n = 25, 28, 33 and 32 mice for vehicle- and tianeptine-treated WT and R6/1 mice, respectively. (**b**) In EPM, there is no significant difference in the locomotor activity between genotypes or between treatments, which is revealed by ambulatory distance. (**c**) In NSF, food consumption was not significantly different between genotypes or between treatments; values are mean ± s.e.m; n = 12, 9, 14, and 13 mice for vehicle- and tianeptine-treated WT and HTT CAG140 mice, respectively. Significance was assessed by two-way ANOVA followed by Tukey's multiple comparison test (**a, b, c**). ****P* < 0.001; *ns*, not significant.

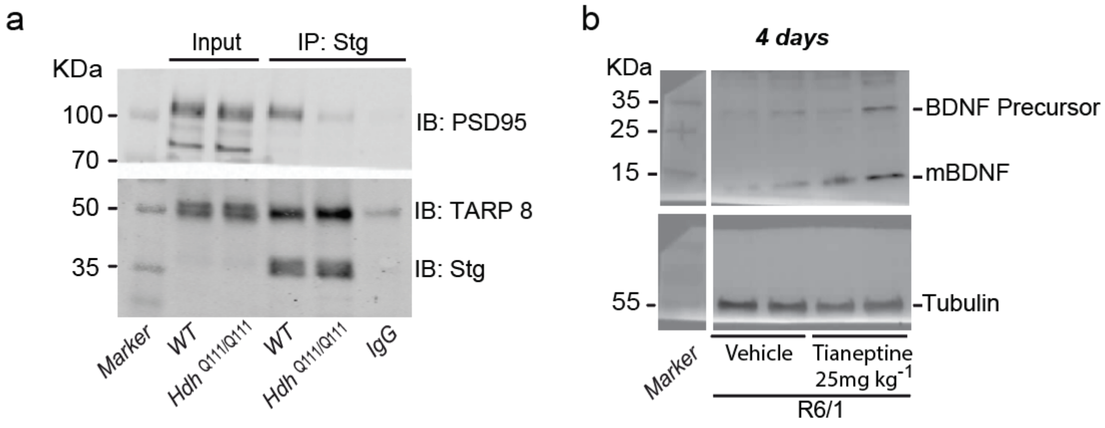

Supplementary Figure 6 Original full blots for Fig. 3a and Fig. 4b. (**a**) Original full blots for Fig. 3a. Co-immunoprecipitation (CO-IP) of PSD95 and stargazin complexes for WT and HdhQ111/Q111 mice. (**b**) Original full blots for Fig. 4b. Mature BDNF (mBDNF) and tubulin were analyzed by immunoblot for R6/1 mice treated with saline (vehicle) or tianeptine (25 mg kg^{-1}, i.p. daily for 4 days).

Supplementary Methods

Immunostaining

For surface GluA2-AMPAR staining, live neurons were incubated with mouse anti-N-terminal GluA2 antibody (a kind gift from E. Gouaux, Oregon Health and Science Unieristy, USA)(1:1000) for 7 minutes followed by fixation and Alexa Fluor 568 goat-anti-mouse secondary antibodies (Thermo Scientific). For total GluA2-AMPAR or HA staining, cells were fixed and permeabilized before the incubation with anti-GluA2 antibody and anti-HA antibody (Cell Signaling Technology) and the following Alexa Fluor 568 secondary antibodies.

Fluorescence Resonance Energy Transfer (FRET)- Fluorescence-lifetime imaging microscopy (FLIM)

A FRET-based CamKIIα, named REACh-CamKII is a kind gift from R. Yasuda (Max Planck Insitute, Florida, USA). The amino and carboxy termini of CamKIIa are labeled with the FRET pair of monomeric enhanced green fluorescent protein (mEGFP) and resonance energy-accepting chromoprotein (REACh), a non-radiative yellow fluorescent protein variant. [1,2]. FLIM experiments were performed at 37 °C using an incubator box with an air heater system (Life Imaging Services) installed on an inverted Leica DMI6000B (Leica Microsystem) spinning disk microscope and using the LIFA frequency domain lifetime attachment (Lambert Instruments, Roden, The Netherlands) and the LI-FLIM software. Cells were imaged with an HCX PL Apo X 100 oil NA 1.4 objective using an appropriate GFP filter set. Cells were excited using a sinusoidally modulated 1-W 477nm LED (lightemitting diode) at 40 MHz under wild-field illumination. Emission was collected using an intensified CCD LI2CAM camera (FAICM; Lambert Instruments). The phase and modulation were determined from a set of 12 phase settings using the manufacturer's LI-FLIM software.

Lifetimes were referenced to a 1µM solution of fluorescein in in Tris-HCl (pH 10) that was set at 4.00 ns lifetime. Signals were recorded with a back-illuminated Evolve EMCCD camera (Photometrics). Acquisitions were carried out on the software MetaMorph (Molecular Devices).

Supplementary References

1. Lee, S.J., Escobedo-Lozoya, Y., Szatmari, E.M. & Yasuda, R. Activation of CaMKII in single dendritic spines during long-term potentiation. Nature **458**, 299-304 (2009).
2. Yasuda, R. Studying signal transduction in single dendritic spines. Cold Spring Harb Perspect Biol **4**(2012).
